# Systematic identification of gene combinations to target in innate immune cells to enhance T cell activation

**DOI:** 10.1038/s41467-023-41792-8

**Published:** 2023-10-09

**Authors:** Lei Xia, Anastasia Komissarova, Arielle Jacover, Yehuda Shovman, Sebastian Arcila-Barrera, Sharona Tornovsky-Babeay, Milsee Mol Jaya Prakashan, Abdelmajeed Nasereddin, Inbar Plaschkes, Yuval Nevo, Idit Shiff, Oshri Yosefov-Levi, Tamara Izhiman, Eleonora Medvedev, Elad Eilon, Asaf Wilensky, Simon Yona, Oren Parnas

**Affiliations:** 1 The Lautenberg Center for Immunology and Cancer Research, Faculty of Medicine, The Hebrew University of Jerusalem, Jerusalem, 91120 Israel; 2 Core Research Facility, Faculty of Medicine, The Hebrew University of Jerusalem, Jerusalem, 91120 Israel; 3 I-CORE Bioinformatics Unit of the Hebrew University and Hadassah Medical Center, Jerusalem, 91120 Israel; 4 Department of Periodontology, Hadassah Medical Center, Faculty of Dental Medicine, Hebrew University of Jerusalem, Jerusalem, 91120 Israel; 5 The Institute of Biomedical and Oral Research, Hebrew University, Jerusalem, 91120 Israel

**Keywords:** Immunology, Immunogenetics, Cancer immunotherapy

## Abstract

Genetic engineering of immune cells has opened new avenues for improving their functionality but it remains a challenge to pinpoint which genes or combination of genes are the most beneficial to target. Here, we conduct High Multiplicity of Perturbations and Cellular Indexing of Transcriptomes and Epitopes (HMPCITE-seq) to find combinations of genes whose joint targeting improves antigen-presenting cell activity and enhances their ability to activate T cells. Specifically, we perform two genome-wide CRISPR screens in bone marrow dendritic cells and identify negative regulators of CD86, that participate in the co-stimulation programs, including *Chd4*, *Stat5b*, *Egr2*, *Med12*, and positive regulators of PD-L1, that participate in the co-inhibitory programs, including *Sptlc2, Nckap1l*, and *Pi4kb*. To identify the genetic interactions between top-ranked genes and find superior combinations to target, we perform high-order Perturb-Seq experiments and we show that targeting both *Cebpb* and *Med12* results in a better phenotype compared to the single perturbations or other combinations of perturbations.

## Abstract (web summary)

Determining targets for genetic engineering of immune cells remains a challenge. Here, we apply genome-wide CRISPR screens with a high-order Perturb-seq (named HMPCITE-seq) to identify gene combinations whose joint targeting improves innate immune cell function.

## Introduction

**Fig. 1:**
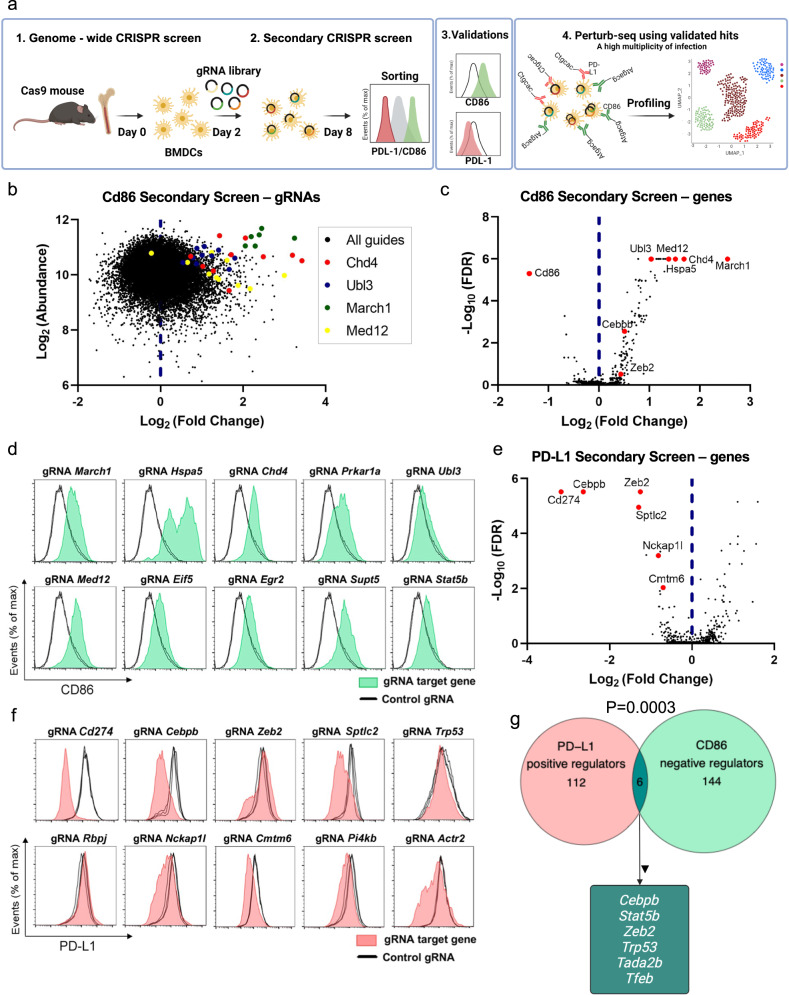
CRISPR screens to identify regulators of CD86 and PD-L1. **a** Workflow of High Multiplicity of Perturbations and Cellular Indexing of Transcriptomes and Epitopes by sequencing (HMPCITE-seq). **b** Enrichment of gRNAs in the secondary screen for regulators of CD86. Log2 fold change of gRNA normalized counts in CD86^High^ sorted cells divided by CD86^Low^ sorted cells (*x* axis). Average abundance of gRNAs normalized counts (*y* axis). **c** A volcano plot showing the results for each gene in the CD86 secondary screen. Log-transformed false discovery rate (FDR) was calculated based on the MAGeCK algorithm (*y* axis). **d** FACS analysis of the top ten negative regulators of CD86 according to the secondary screen. Plots are ordered according to the gene ranking. In each plot, CD86 levels of targeted genes are colored in green, and two non-targeting gRNAs in black. For each gene a representative graph of 3–6 experiments using two different gRNAs, is shown. **e** A volcano plot, similar to (**c**) showing the results for each gene in the PD-L1 secondary screen. **f** FACS analysis of the top ten positive regulators of PD-L1 according to the secondary screen. Plots are ordered according to the gene ranking. In each plot, PD-L1 levels of targeted genes are colored in red, and non-targeting gRNAs in black. For each gene a representative graph of a total of 3–6 different experiments using two different gRNAs, is shown. **g** Overlap of top-ranked genes in the CD86 and PD-L1 secondary screens (*p* value was calculated using a hypergeometric test). Source data are provided as a Source Data file.

Cellular therapy, including adoptive cell transfer (ACT), in which specific cell populations are selected and expanded ex vivo before being transferred back to the patient, has been shown to be a beneficial cancer treatment^1,2^. A deeper understanding of immune cell circuits and more advanced tools for gene targeting have substantial potential to enhance ACT’s effective immune response against tumors. Progress has been achieved by demonstrating that clustered regularly interspaced short palindromic repeats (CRISPR) can be safely used to target genes in immune cells^3^, thereby possibly improving immune cell function in the hostile tumor microenvironment (TME). Pooled genome-scale screens may be used to discover a set of genes that when perturbed individually affect a specific cell-intrinsic readout, such as protein expression, cell proliferation, or survival^4–8^. Conversely, Perturb-seq screens, which combine pooled screening with single-cell RNA-seq (scRNA-seq) or CITE-seq readouts, have thus far been mostly limited to hundreds of perturbations, pre-selected before the screening^9–14^. Although technically feasible^15^, genome-scale Perturb-Seq requires millions of cell profiles for statistical power^12^, and thus remains very costly, even when leveraging newer and potentially cheaper sequencing technologies^16^. Moreover, individual gene perturbations may not be sufficient to achieve a desired outcome, and because combinations of perturbations can have non-additive effects (genetic interactions), desired combinations cannotyet be predicted solely from the impact of individual perturbations. Finding genetic interactions by targeting pairs of genes in the same cell, thus revealing combinations of genes to target, is not yet possible at a high scale. Here, we address these challenges, using two previously established technologies, CRISPR screens and Perturb-seq, to discover combinations of genes to target and improve a desired cellular function (Fig. 1a). We name the platform High Multiplicity of Perturbations and Cellular Indexing of Transcriptomes and Epitopes by sequencing (HMPCITE-seq) and we apply this method to rewire the circuits in innate immune dendritic cells.

Dendritic cells are antigen-presenting cells (APCs) that bridge between the innate and adaptive immune systems and play a critical role in T-cell priming. Capturing tumor cells, processing them, and presenting relevant tumor-derived peptides in the correct context are essential for the mounting of anti-tumor immune response. However, tumor-associated cells secrete inhibitory cytokines including IL-6 and IL-10 that restrict monocyte differentiation to monocyte-derived-dendritic cells (mo-DCs), concomitantly inducing the recruitment and differentiation of suppressive monocyte-derived cells. Preventing the accumulation of suppressive innate immune cells in the TME, and increasing the amount and activity of DCs, can induce an effective immune response^17–19^. Thus, the balance between immune cell states is critical for the outcome of diseases.

We use HMPCITE-seq to rewire the circuits in DCs controlling two key and opposing functionalities important for T-cell activation by DCs: CD86, a receptor that is expressed on the DC’s surface and interacts with the T-cell receptor CD28^20, 21^, is essential for priming and clonal expansion of T cells^22^ and may also play a role in effector T-cell activation in peripheral organs;^23^ and PD-L1, whose expression attenuates T-cell priming and activation.

We perform a genome-wide CRISPR screen in primary bone marrow-derived dendritic cells (BMDCs) using CD86 and PD-L1 as readouts, followed by secondary screens and validations with individual guide RNAs (gRNA) (Fig. 1a). We then target 11 validated hits in a high multiplicity of infection (MOI) using single-cell RNA-seq (scRNA-seq) readouts, to find combinations of perturbations to enhance immune cell function.

## Results

### Genome-wide CRISPR screens to find regulators of CD86 and PD-L1 reveal shared validated targets

We reasoned that CD86 and PD-L1 would each represent a meaningful and opposing functional readout for DCs’ ability to prime and activate T cells. We initially searched for genes that modulate CD86 levels by using BMDCs as a model of APCs that can cross-present antigens and induce both CD8 T-cell and CD4 T-cell priming^24^, similar to primary DCs. We recovered bone marrow cells from mice that constitutively expressed *Cas9*^25^, infected the cells with a gRNA library^26^, and six days later sorted CD11c-positive cells according to CD86 expression levels (Fig. 1a, “Methods”). As expected, CD86 had the highest ranking among the positive regulators (Supplementary Data 1). To identify false positives, while applying an initial lenient threshold, we performed a secondary screen that included 10 additional gRNAs (Methods), targeting the top 2000 negative ranked genes and top 500 positive ranked genes,  and searching mainly for targeted genes that increase the levels of CD86 (Fig. 1b, c). We observed lower false discovery rates (FDRs) in the secondary screen compared to the genome-wide screen (Supplementary Fig. 1a, Supplementary Data 2) and a high correlation between different approaches to screen data analysis, supporting the robustness of our results (Supplementary Fig. 1b). Validations using individual gRNAs showed an increase in CD86 expression following the targeting of each of the ten top-ranked negative regulators (Fig. 1d, Supplementary Fig. 1c), including *March1* (ranked #1), an E3 ubiquitin ligase that was previously shown to regulate CD86 cell membrane expression^27^. Targeting *Ubl3* (ranked #5), a ubiquitin-like protein that was recently found to interact with *March1*^28^, also increased CD86 levels (Fig. 1d). Other validated top genes included the chromatin regulator *Chd4* (ranked #3) which encodes a subunit of the nucleosome remodeling and deacetylase (NuRD) complex, and two subunits of the mediator complex, MED12 (ranked #6) and MED30 (ranked #12) (Fig. 1d and Supplementary Fig. 1d). Overall, 13 subunits of the mediator complex were among the top 80 ranked negative regulators (Supplementary Data 2, FDR < 0.05).

CD86 induction is associated with DC maturation and may therefore be coupled with a simultaneous increase in the expression of inhibitory receptors and cytokines, thus avoiding an uncontrolled immune response that damages intact tissue. Indeed, toll-like receptors (TLRs) agonists or interferon increase the expression of CD86 but also of PD-L1 (Supplementary Fig. 1e), the former inducing T-cell activity and the latter restricting it. The simultaneous induction of co-stimulatory and co-inhibitory receptors following ex vivo manipulation can limit these cells’ ability to induce an immune response in vivo.

To search for genes that attenuate the inhibitory program, we performed an additional genome-wide CRISPR screen in BMDCs with PD-L1 expression as a readout, followed by two repeated secondary screens (Supplementary Fig. 1f, “Methods”). Genetic screens for PD-L1 regulators were previously done in cell lines^29,30^ but not in primary immune cells. *Cd274*, which encodes PD-L1, was the top-ranked positive regulator (Fig. 1e, Supplementary Data 3 and 4). The top-ranked genes were also enriched for members of the immune response-regulating cell surface receptor signaling pathway (FDR = 0.00017, Supplementary Fig. 1g). Individual gRNA experiments verified the phenotype of *Cmtm6* (ranked #8), a known regulator of PD-L1 recycling and transport to the cell membrane in a pancreatic tumor cell line^31,32^ (Fig. 1f, Supplementary Fig. 1h). Targeting *Sptlc2* (ranked #4), which encodes an enzyme that initiates the biosynthesis of sphingolipids, reduced the expression of PD-L1. Myriocin, a drug that inhibits SPTLC2, and CRISPR-targeted *Sptlc2*, had a similar effect (Supplementary Fig. 1i). Other top-ranked genes included five subunits of ARP2/3, actin branching complex^33^, and its associated WAVE complex (Supplementary Data 4), which have a role in DC migration^29,30^, but were not known to affect PD-L1 levels. Targeting a subunit of the WAVE complex, *Nckap1l* (ranked #7), which encodes HEM1, reduced PD-L1 (Fig. 1f). Interestingly, patients harboring mutations in NCKAP1L suffer from extensive lymphocyte proliferation^34,35^. The effect of mutant *NCKAP1L* on the clinical outcome may be actin-independent^35^, and our results suggest that a reduced level of PD-L1 may contribute to patients’ lymphocyte proliferation.

Overall, we identified 150 negative regulators of CD86 (Pval < 0.05, ten tested and validated individually) and 118 positive regulators of PD-L1 (Pval < 0.05, ten tested individually), and more than half of them were not previously reported in these roles. We found six overlapping genes between the screens (Fig. 1g, Pval = 0.0003 hypergeometric test). These included: *Zeb2* (ranked #3 positive regulator in the PD-L1 screen, and #108 negative regulator in the CD86 screen), *Stat5b* (ranked #35 positive regulator in the PD-L1 screen, and #10 negative regulator in the CD86 screen), and *Cebpb* (ranked #2 positive regulator in the PD-L1 screen, and #52 negative regulator in the CD86 screen) (Supplementary Data 2 and 4). Targeting these genes may rewire BMDCs to induce the activation program and restrict the suppressive program.

### *Cebpb*-targeted BMDCs enhance T-cell priming and restrict tumor growth

We first focused on CEBPB, an enhancer-binding protein that is involved in reprogramming and cancer development^36^, and previously described as a pioneer factor of innate immune cell differentiation^37^. The *Cebpb* transcript can be translated from three different starting sites to form lap*, lap, or lip isoforms^38^. Transduction with lentiviruses that encode gRNAs, which align 3’ to the translation start-sites of each of the three isoforms of *Cebpb*, elevated CD86 levels and reduced PD-L1 levels (Supplementary Fig. 2a, b). Consistent with a previous study^39^, BMDCs recovered from a *Cd11c*-Cre *Cebpb*(fl/fl) mouse, expressed higher CD86 and MHC-II levels compared to control BMDCs (Supplementary Fig. 2c–f). To further explore the function of *Cebpb*-targeted BMDCs, we followed with in vivo and in vitro experiments.

**Fig. 2:**
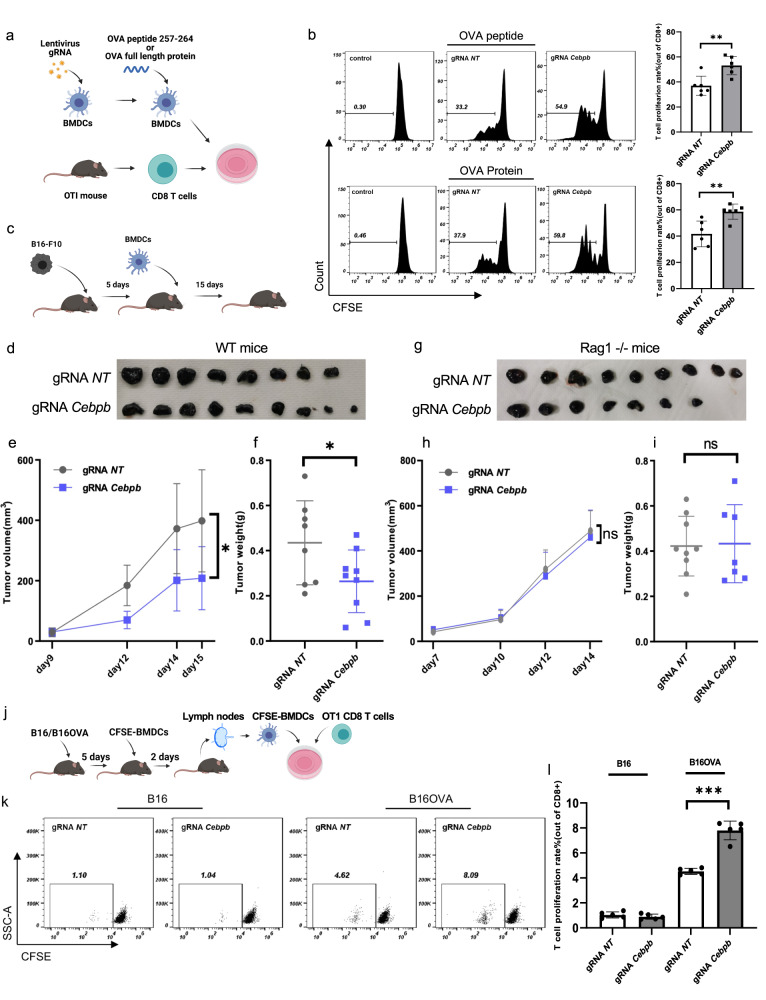
Targeting *Cebpb* in BMDCs improves T-cell priming and reduces tumor growth. **a** Workflow of the experimental setting. **b** FACS analysis of CFSE-positive cells showing T-cell proliferation. For the experiment in the upper panel OVA peptide (257–264) was added to the BMDCs, and in the lower panel OVA full-length protein was added to the BMDCs (*p* = 0.0039 in the upper and *p* = 0.0064 in the lower panel, two-tailed unpaired *t*-test, *n* = 6 independent samples). **c** Workflow of the experimental setting in vivo. **d**, **g** Photos of B16 tumors from C57BL/6J mice (**d**) or C57BL/6J Rag1^−/−^ mice (**g**). **e**, **h** The graphs show tumor volume *V* = (*W* ^2^ × *L*)/2. C57BL/6J mice were used, in (**e**) (*p* = 0.0186, unpaired, two-tailed *t*-test, *n* = 9 mice), and C57BL/6J Rag1^−/−^ mice in (**h**) (*n* = 9 mice). **f**, **i** Tumor weight was measured at the end of the experiment from the same mice that are shown in (**e**) and (**h**), respectively. In (**f)** *p* = 0.047, two-tailed unpaired *t*-test. **j** CFSE-labeled gRNA-NT or gRNA*-Cebpb* BMDCs were injected to the tumor site. Two days later, the CFSE-positive cells were sorted from the lymph node (LN) of tumor-bearing mice and co-cultured with CFSE-labeled OT-I CD8 T cells. **k** In the left two panels B16 cells were used and in the right two panels B16-OVA cells were used. *n* = 5 independent samples. **l** Quantification of the experiment that is shown in (**k**) (*p* = 0.0003, two-tailed unpaired *t*-test). Data are presented as mean values ± SD in all the graphs (***P < 0.001, **P value < 0.01, *P value < 0.05). Source data are provided as a Source Data file.

DCs capture antigens, process them, migrate to the lymph nodes (LNs), and present related peptides on MHC molecules along with the expression of additional receptors and cytokines that initiate T-cell clonal expansion and transition to an effector state. To examine whether *Cebpb*-targeted BMDCs can enhance T-cell priming, we used gRNA-*Cebpb* and gRNA-non-targeting BMDCs (gRNA-NT), and measured CD8 T-cell proliferation. We also included BMDCs that overexpress CD86 and found that overexpression of CD86 improved OT-I T-cell proliferation, pointing to CD86 as a rate-limiting factor of T-cell priming (Supplementary Fig. 2g–i). The enhanced priming by gRNA-*Cebpb* BMDCs was seen both when the OVA peptide was used or when the protein was used, showing that this result is also valid when antigens should be internalized and processed (Fig. 2a, b).

To examine the effect of *Cebpb*-targeted BMDCs on tumor development in vivo, B16 cells were injected subcutaneously, and five days later, gRNA-*Cebpb* or gRNA-NT BMDCs were injected into the tumor site (Fig. 2c, “Methods”). Based on tumor size and weight, the targeting of gRNA-*Cebpb* in BMDCs restricted tumor growth (Fig. 2d–f). These modified BMDCs did not affect tumor growth in tumor-bearing *Rag* mutant mice (Fig. 2g–i). Thus, the restriction of tumor growth by the modified BMDCs is mediated by the host adaptive immune system. To further test BMDC function in vivo, we injected B16 or B16-OVA cells into mice, and five days later we introduced labeled BMDCs that were CRISPR targeted for *Cebpb*, for 48 hours. We then isolated the labeled BMDCs from tumor-adjacent LN and incubated them with OT-I CD8 T cells. gRNA-*Cebpb* BMDCs recovered from the LN of B16-OVA, but not from the LN of B16 tumor-bearing mice, were superior in activating OT-I T cells in vitro (Fig. 2j–l) compared to gRNA-NT BMDCs. Based on these results, we conclude that gRNA-*Cebpb* BMDCs can capture tumor antigens in vivo, process tumor antigens, migrate to the LNs, and have an increased capability to induce T-cell priming.

gRNA-*Cebpb*-targeted DCs also had improved migration ability (Supplementary Fig. 2j), with similar phagocytic capability (Supplementary Fig. 2k). Thus, targeting *Cebpb* does not affect DC maturation directly but improves the ability of the cells to prime T cells. Taken together, in addition to their superior CD8 T cells priming in vitro, *Cebpb*-targeted BMDCs demonstrated functional superiority in restricting tumor growth in vivo.

### The effect of *Cebpb* on cell state and inflammatory response is dependent on *Nr4a3*

**Fig. 3:**
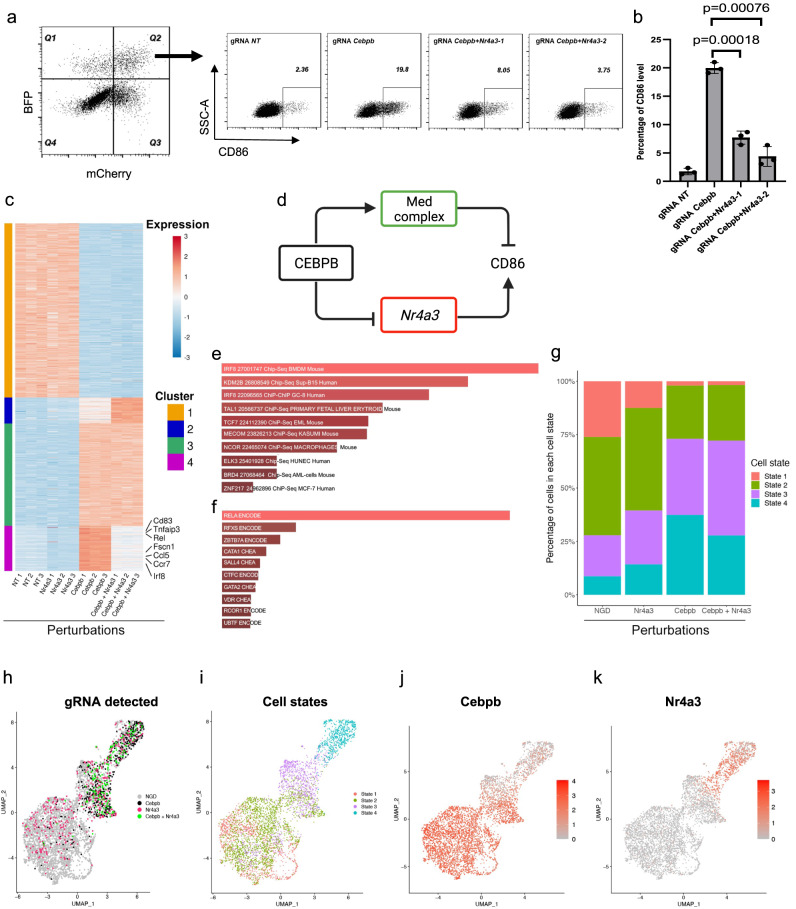
Genetic interactions between *Cebpb* and *Nr4a3*. **a** The expression of CD86 in *Cebpb* and *Cebpb Nr4a3* targeted cells. Cells were gated according to the expression of mCherry and BFP (left plot). Two different gRNAs were used to target *Nr4a3*. **b** Quantification of the experiment that is shown in (**a**). The y axis shows the percentage of CD86 positive cells (two-tailed unpaired *t*-test. *n* = 3 independent samples). Data are presented as mean values ± SD. Source data are provided as a Source Data file. **c** RNA-Seq experiment that included cells with different gRNAs. For each gRNA, *n* = 3 independent samples. Differentially expressed genes between cells that carry gRNAs-*Cebpb* and gRNAs-NT are shown in the heatmap. K-mean clustering (*k* = 4) was applied, and the expression value was Z-score standardized across different treatments. **d** Cartoon showing genetic interactions with *Cebpb*. **e** Enrichment analysis of genes that were assigned to cluster 3 of the heatmap that is shown in (**c**), source data are provided as Supplementary Data 8. **f** Enrichment analysis of genes that were assigned to cluster 4 of the heatmap that is shown in (**c**), source data are provided as Supplementary Data 8. **g–k** ScRNA-seq of cells that were infected with gRNA-*Cebpb* and gRNA-*Nr4a3*. 4740 cells are presented**. g** Percentages for each cell state are shown on the *y* axis. **h** Uniform manifold approximation and projection (UMAP) showing which gRNAs are expressed by each cell. NGD, no guide detected. **i** UMAP showing the distribution of cell states. **j** UMAP is colored according to the normalized expression of *Cebpb* mRNA in different cells. **k** UMAP is colored according to the normalized expression of *Nr4a3* mRNA in different cells.

To explore additional phenotypic features of *Cebpb*-targeted BMDCs, we first tested the expression of additional proteins that regulate T-cell proliferation. TNF alpha, IL-12, MHC-II, and CD80 expression were increased in *Cebpb*-targeted cells (Supplementary Fig. 3a), showing that *Cebpb* regulates additional genes other than *Cd86*. To further investigate the effect of gRNA-*Cebpb*, we performed bulk RNA-seq (“Methods”) and found that *Cebpb* knockout (KO) upregulated 383 genes, including *Cd86* and genes that mediate T-cell activation and DC migration. In addition, *Cebpb* KO downregulated 438 genes including the pattern recognition receptors *Marco* and *Mrc1* (Supplementary Data 5, log2fc > 0.5, FDR < 0.05, among highly expressed genes). Some of the differentially expressed genes (DEGs), such as *Med14* (Supplementary Fig. 3b), were also top-ranked negative genes in the CD86 CRISPR screens, suggesting that their reduced expression in *Cebpb*-targeted cells mediate the effect on CD86 level (Fig. 3d).

*Nr4a3*, which encodes a nuclear receptor, was one of the top DE transcription factors, with high expression in gRNA-*Cebpb*-targeted cells (log2fc = 1.75, FDR = 9.68 × 10^−109^, Supplementary Fig. 3b, c). ChIP-seq experiments using anti-CEBPB antibodies confirmed that CEBPB binds directly to the *Nr4a3* locus (Supplementary Fig. 4, Supplementary Data 6), and ATAC-seq experiments showed increased chromatin accessibility at the *Nr4a3* locus in *Cebpb*-targeted cells (Supplementary Fig. 4f, g, Supplementary Data 7). To explore if CD86 expression in *Cebpb*-targeted cells depends on *Nr4a3*, BMDCs were co-transduced with lentivectors encoding mCherry-gRNA-*Nr4a3* and BFP-gRNA-*Cebpb*. Indeed, CD86 induction in the *Cebpb*-targeted cells was dependent on *Nr4a3* (Fig. 3a, b). We next performed RNA-seq of cells that expressed either one of the gRNAs or both gRNA-*Nr4a3* and gRNA-*Cebpb*. DEGs between gRNA-*Cebpb* and gRNA-NT were partitioned to four different clusters (Fig. 3c, Supplementary Data 8). Cluster 3 included genes that had similar expression in the double KO of gRNA-*Nr4a3* gRNA-*Cebpb* and in the single KO of gRNA-*Cebpb*, and these genes were enriched for IRF8 binding sites according to published ChIP-seq data (Enrichr FDR 5.1 × 10^−7^, Fig. 3e). However, the expression of 125 genes in cluster 4 was dependent on *Nr4a3* and these genes were enriched for RELA binding sites according to published ChIP-seq data^40^ (Enrichr FDR 1.3 × 10^−7^, Fig. 3f). The genes in cluster 4 included *Irf8*, *Cd83*, *Ccl5*, *Rel*, *Tnfaip3*, and in addition *Ccr7* and *Fscn1*, which are key markers of a subset of DCs, mregDCs^41^/PDL2 positive cells^42, 43^ (“Discussion”).

BMDCs include several subpopulations of cells. Since *Cebpb* was shown to affect the cell state^12,14^, we aimed to investigate if *Nr4a3* controls the BMDCs cell-state distribution and conducted a perturb-seq experiment^12–14,44^. BMDCs were transduced with vectors that encode gRNA-*Cebpb* and gRNA-*Nr4a3*, and six days after the infection, scRNA-seq was performed. As a control, we pursued a parallel experiment in which we transduced cells with lentiviruses that encode gRNA-*Cd274*, gRNA-*Cd86* or gRNA-NT. We regressed the effects of cell cycle (Supplementary Fig. 5a, b, “Methods”) and defined four cell states (Supplementary Fig. 5c–m). In this experiment, we included barcoded anti-CD86 and anti-PD-L1 antibodies (antibody-derived tags, ADT) and detected a significant reduction in the protein levels of CD86 and PD-L1 in cells that contained the matched gRNA (Supplementary Fig. 6a–d). Cells containing either gRNA-*Cd274* or gRNA-*Cd86* were uniformly distributed across the four cell states, suggesting that expression of PD-L1 or CD86 per se, and infection with lentivirus do not affect the cell state (Supplementary Fig. 6e–j).

In the bone marrow culture that was infected with gRNA*-Cebpb* and gRNA*-Nr4a3*, 449 cells expressed gRNA*-Cebpb*, 400 cells expressed gRNA*-Nr4a3*, and 169 expressed both (Fig. 3h). Cells that were transduced with a lentivirus that encoded gRNA-*Cebpb* were highly enriched in state 4 (Fig. 3g).

Although MHC-II expression was higher in *Cebpb*-targeted cells in state 2, most of the effects of *Cebpb* KO were related to the distribution of cells across cell states, and not on DEGs within a cell state (Supplementary Fig. 6k–m). Cells expressing both gRNA*-Cebpb* and gRNA*-Nr4a3* had a lower percentage of state 4 cells compared to single gRNA-*Cebpb* infected cells (Fig. 3g). The expression of *Cebpb* in state 1 cells and the expression of *Nr4a3* in states 3 and 4 cells (Fig. 3i–k), suggests that *Cebpb* plays a role in the differentiation and survival of state 1 cells and that *Nr4a3* regulates gene expression of mature and mregDCs (states 3 and 4) (Fig. 3).

Next, we tested the effect of *Cebpb* on primary mature innate immune cells. CD11c-positive cells were isolated from the spleen of three *Cd11c*-Cre *Cebpb*(fl/fl) mice and three control mice and scRNA-seq experiment was performed (Supplementary Fig. 7). We identified the different subsets of myeloid cells and DCs, and observed cells that showed a similarity to state 1 BMDCs and had low expression of MHC-II. The percentage of those cells was almost half in *Cd11c*-Cre *Cebpb*(fl/fl) mice samples in comparison to control mice (cluster 7 and cluster 9, 6.5% compared to 12.1% in the control, Supplementary Fig. 7). We also detected an increase in the expression level of CD86 in cDC1 and cDC2 (Supplementary Fig. 7h, i). Similar to *Cebpb* perturbed BMDCs, the expression of *Nr4a3*, and also the expression of the interferon-related genes *Ifi44* and *Ifi44l*, had an inverse correlation with *Cebpb* (Supplementary Fig. 7 k–m, Supplementary Data 9).

Thus, CEBPB is a pioneer transcription factor that affects dendritic cell states, in accordance with previous reports^12,14^, and the genes that are being regulated by *Cebpb* mark mregDCs^41^/PDL2 positive cells^42^. In addition, CEBPB restricts *Nr4a3* expression in BMDCs and splenocytes, and the effect of *Cebpb* KO on gene expression, cell state, and CD86 level, partially depends on intact *Nr4a3*.

### Targeting human monocytes

Tumor-associated DC subtypes and states from mice and humans have similar expression profiles^43^. We next examined the effect of *CEBPB* targeting in human monocytes. We isolated CD14-positive monocytes from the peripheral blood of healthy subjects and electroporated the monocytes with the CAS9 protein and a gRNA. We differentiated the cells and found that after targeting *CEBPB* (Supplementary Fig. 8a, b, "Methods"), the level of CD86 increased and that the expression of other co-regulated genes, such as *CCR7*, *CD80*, *FSCN1*, and *NR4A3, (*Supplementary Fig. 8d, Supplementary Data 10) increased as well, similar to mouse cells (Supplementary Fig. 8c, Supplementary Data 8). Gene annotation analysis showed that overlapping DEGs between mice and humans included immune-related categories such as up-regulation of genes that are involved in cell migration and adhesion (Supplementary Fig. 8e–h). Thus, despite the differences in the origin of cells and in the CAS system delivery method, a partial overlap of DEGs was observed (Supplementary Fig. 8e–i), exemplifying the robustness of our findings and the need to further examine the translational potential.

### *Stat2, Mitf*, and *Rxra* mediate the effect of *Cebpb* on dendritic cell activation

To further investigate how *Cebpb* regulates immune activation, we performed a genome-wide CRISPR screen (Supplementary Data 11) and a smaller scale screen targeting 661 *Cebpb* DEG, using CAS9 *Cd11c*-Cre *Cebpb*(fl/fl) bone marrow cells (Supplementary Fig. 9a, Supplementary Data 12). The smaller scale screen was also done in control mice that expressed *Cebpb* (Supplementary Fig. 9b, Supplementary Data 12). These screens confirmed that *Nr4a3* mediates the increase in CD86 in *Cebpb*-targeted cells.

In addition, we found that the STAT2 binding motif was exposed in *Cebpb*-targeted cells according to the ATAC-seq data (Supplementary Data 13), that the expression of *Stat2* increased in those cells (Supplementary Data 5), and that *Stat2* is found to be a positive regulator of CD86 according to the CRISPR screens results (Supplementary Data 12). Interestingly, *Rxra* had exactly the opposite phenotypes. These data confirm the pivotal role of *Stat2* in activating DCs and strongly support the possibility that in the presence of CEBPB, RXRA restricts DC activation (consistently, this effect is not seen in *Cebpb* KO BMDCs, Supplementary Fig. 9a). Applying motif enrichment analysis (HOMER), we found that CEBPB binds to chromatin regions that are enriched with the FOSL2 and MITF binding motifs (Supplementary Data 14). The screen results show that *Fosl2* is a negative regulator of CD86 and therefore may cooperate with CEBPB to restrict DC activation, while *Mitf* is a positive regulator and a possible competitor of CEBPB. Consistent with this possibility, the ranking of *Mitf* as a positive regulator was much higher in the screen that was performed in the *Cebpb* KO cells (Supplementary Fig. 9).

These findings reveal the mechanism that governs the effect of *Cebpb* in immune cell regulation and the possible role of *Stat2, Mitf*, and *Rxra* in addition to *Nr4a3*.

### Expression programs of perturbed cells are distinct and enhance MHC-I expression

Based on our findings, *Cebpb* is a promising candidate for therapeutic targeting as its perturbation can enhance the immune response and restrict tumor growth. Multiple genes that affect CD86 or PD-L1 were found in the screens (Fig. 1) and targeting them may differentially affect the cell’s expression program. We thus hypothesized that combinations of targeted genes may have a stronger effect compared to single perturbations. Such combinations may broadly induce the activation program while restricting the suppressive program of innate immune cells. We thus aimed to (i) discover how perturbations of validated targets affect the expression program, and (ii) reveal combinations of perturbations that improve the function of innate immune cells.

**Fig. 4:**
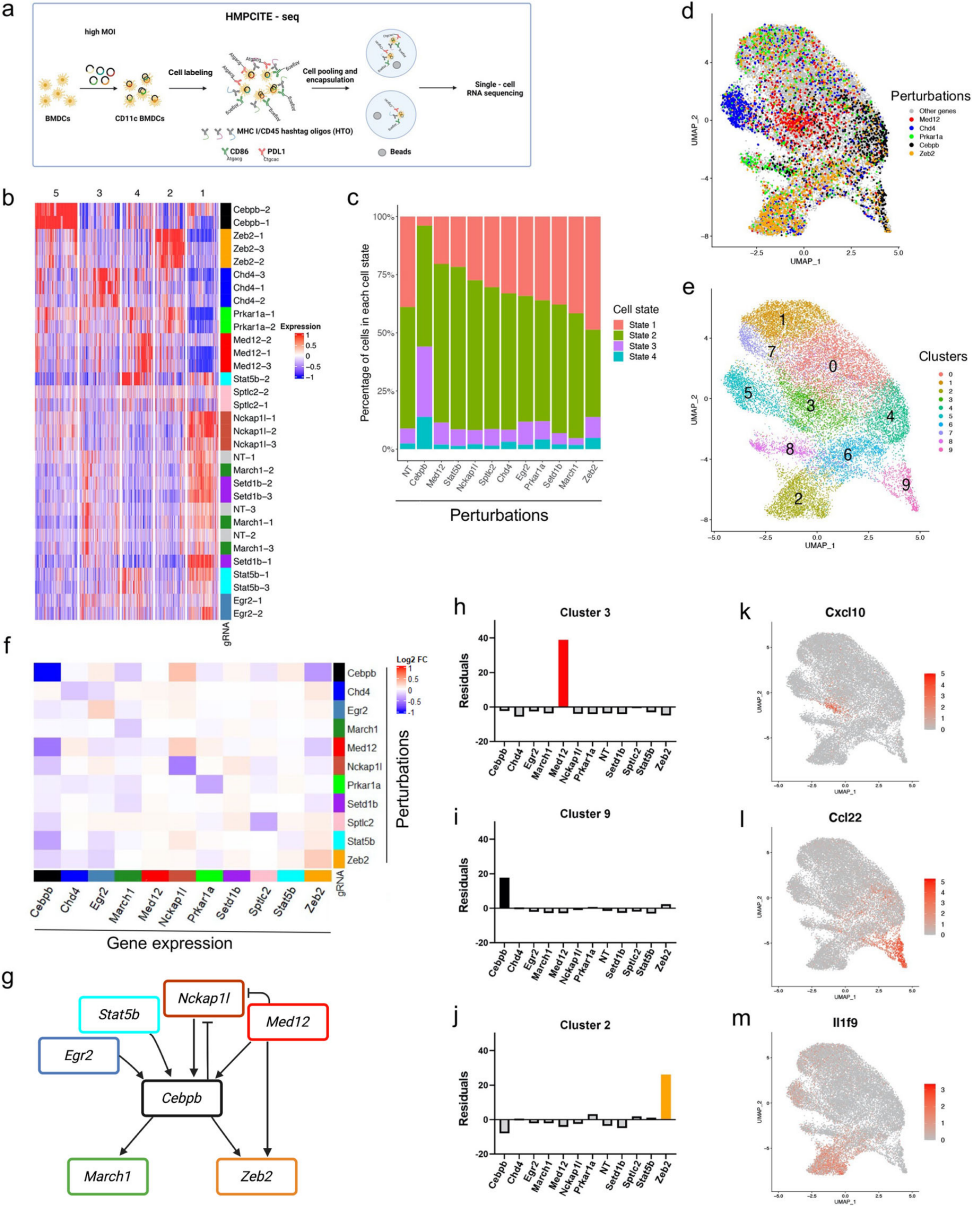
The effect of key regulators on the transcription program. **a** Cells were infected with the pool of selected gRNAs at a high multiplicity of infection (MOI). CD11c-positive cells were sorted and stained with oligo-conjugated anti-CD86, anti-PDL1, and anti-MHC-I/CD45 hashing antibodies. Finally, cells were pooled together for single-cell RNA sequencing. 21,922 cells were included in the analysis. Source data are provided as Supplementary Data 16 and 17. **b** The expression of top variable genes. Each row represents a different gRNA, each column represents a gene. Only cells that express a single gRNA were included. Normalized UMIs were averaged for all the cells that expressed the same gRNA, and columns were Z-score standardized. Rows are hierarchically clustered, and columns are K-means (*k* = 5) clustered. **c** The distribution of cells with different gene perturbations across the four cell states. **d** Uniform manifold approximation and projection (UMAP). Cells are colored by the identified gRNA. Selected gRNAs are shown. **e** Different clusters that were identified based on RNA expression. Cluster numbers are shown. **f** The effect of each knockout on the expression of the selected genes. Rows represent the perturbed gene according to the gRNA that was detected, and columns represent the gene expression. Fold-changes relative to cells with non-targeting-gRNAs are shown. **g** Genetic interactions across targeted genes. **h-j** Gene perturbations enrichment in clusters. The standardized residual values of a chi-squared test are shown (*y* axis). (**h**) Cluster 3, (**i**) Cluster 9 (**j**) Cluster 2. **k–m** UMAPs showing the normalized expression of selected genes in clusters 3, 9 and 2. (**k)** Cxcl10, (**l)** Ccl22, (**m)** Il1f9.

We performed the last step of HMPCITE-seq, using a pool of 32 gRNAs that target 11 genes that were validated as regulators of CD86 or PD-L1 (Fig. 4a). BMDCs were infected at MOI of 1.6, to include in the same experiment cells that express single or double gRNAs (Supplementary Fig. 10a–f). CD11c-positive cells were sorted and incubated with oligo-conjugated anti-CD86 and anti-PD-L1 antibodies, as well as HashTag-Oligos (HTOs) antibodies (anti-MHC-I/CD45 conjugated to different barcodes)^45,46^. This design supports the exploration of expression changes in the perturbed cells, the genetic interactions within the set of targeted genes, and the effect of the perturbations on the expression of CD86, PD-L1, and MHC-I mRNA and protein.

HTOs allowed us to distinguish between multiple guides that were transduced to one cell and multiple cells (each with one guide) that entered the same droplet during single-cell barcoding. We recovered 36,286 high-quality cell profiles and retained from them 21,922 cell profiles with a single HTO that expressed one or two guides (Supplementary Fig. 11c and Supplementary Data 15). gRNAs that target the same gene had a similar impact on expression (Fig. 4b), showing the robustness of the experimental setting. Notably, cells perturbed with *March1*, *Setd1b*, *Nckap1l*, *and Sptlc2* gRNAs clustered with those from cells receiving non-targeting guides and had a low number of DEGs (Supplementary Data 16). This may indicate that the effect of these gene perturbations is post-transcriptional.

Cells expressing gRNA that target *Cebpb*, *Zeb2*, *Chd4*, *Med12*, and *Prkar1a* were clustered according to the detected perturbed genes (Fig. 4d), showing a dominant effect of the perturbation and the high efficiency of gRNA detection. Perturbations of *Cebpb*, *Zeb2*, *Chd4*, *Stat5b*, *Egr2*, *Med12*, and *Prkar1a* increased the expression of genes that are involved in antigen processing and presentation, such as *B2m* (GO:0002428, FDR = 1.92e–06) (Supplementary Fig. 10h, and Supplementary Data 16 cluster 3). The effects on genes that were not used as readouts in the screens, yet are related to antigen presentation, show that choosing two receptors as genetic screen readouts is a valid approach for finding regulators of a broad cellular response.

Consistent with the experiment shown in Fig. 3, *Cebpb-*targeted cells were enriched in state 4. In addition, *Zeb2*-targeted cells had an elevated fraction of cells that expressed markers of state 1, while *Med12-* and *Stat5b-*targeted cells had an elevated fraction of cells that expressed markers of state 2 (Fig. 4c and Supplementary Fig. 10g, m–q).

To achieve a mechanistic insight and to find genes that mediate the effect of the set of KO genes, we compared DEGs found in the Perturb-seq experiment with the list of top-ranked genes of the CRISPR screens. KO of *Cebpb* increased the expression of *Irf4*, which was the top-ranked negative regulator in the PD-L1 screen, raising the possibility that the increase in *Irf4* expression mediates changes in PD-L1 and CD86 levels in *Cebpb*-targeted cells. In addition, targeting *Chd4*, *Med12*, *Zeb2*, *Prkar1a*, or *Cebpb* reduced the expression of ten genes that were found as negative regulators in the CD86 CRISPR screen (Supplementary Fig. 10i), including *Slfn2* which is known to be involved in proper immune responses^47, 48^. These results suggest that a reduced level of *Slfn2* elevates the level of CD86 and mediates innate immune activation and that this pathway is regulated by *Cebpb*, *Med12*, and *Prkar1a* as well. We profiled the cross-regulation between top-ranked genes in the screens and found that some of the top-ranked genes internally regulate each other’s expression (Fig. 4f, g, Supplementary Fig. 10i–l). Interestingly, these analyses revealed that *Cebpb* expression is altered in *Med12*, *Stat5b* and *Egr2*^49^ targeted cells.

Our data indicate a partial redundancy in the genetic circuits that control the expression of CD86 and PD-L1. However, cells that had different perturbations were grouped separately (Fig. 4d). For example, cluster 3 associated cells were enriched for gRNA-*Med12* and expressed an elevated level of interferon-related genes including *Ifit1*, *Ifit2*, *Ifit3* and *Isg15*, and *Cxcl10* that promote the recruitment of CD8 T cells^50^, while *Ccl22* and *Il36g* (ILIF9) were enriched in cluster 9 and 2, respectively (Fig. 4e, h–m, Supplementary Data 16, 17, “Methods”). Other prominent cytokines and receptors that regulate T cells and were differentially expressed in specific clusters included MHC molecules, *Il18*, and *Inhba*^51^ (Supplementary Fig.10m–t).

Based on this analysis we aimed to find combinations of genes that produce a desired mix of signals which support immune activation.

### Targeting both *Med12* and *Cebpb* enhances DC activation

To find an effective combination of genes to target, we first explored the effect of each targeted gene on protein expression, which was obtained based on ADT and HTO sequencing data. We detected a significant (*t*-test, FDR < 0.05, compared to gRNA-NT) increase in the protein level of CD86 in cells that express gRNA that target *Med12, March1, Prkar1a, Cebpb*, *Egr2*, *Chd4*, and a strong reduction of PD-L1 protein levels in cells that express gRNA that target *Stat5b*, *Cebpb*, *Nckap1l*, *Med12*, and *Chd4* (Supplementary Fig. 11a, b), consistent with FACS validations of the CRISPR screen results.

**Fig. 5:**
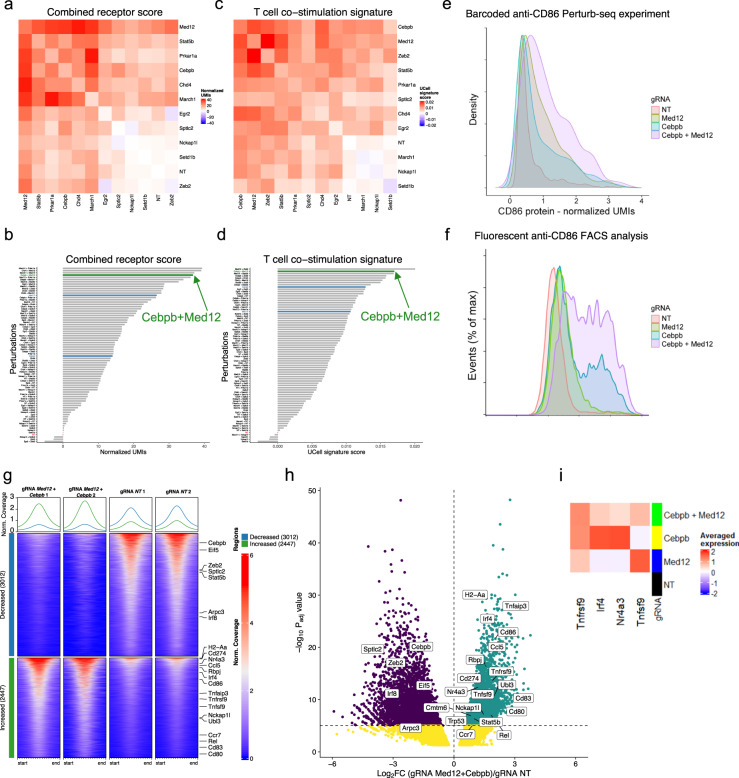
Combinations of targeted genes alters the expression of co-stimulatory molecules and gene expression. **a** A combined score of antibody-derived tags (ADTs) of CD86, PD-L1, and MHC-I/CD45. Protein score was calculated as the sum of CD86 and MHC-I/CD45 normalized UMIs minus PD-L1 normalized UMIs. For each perturbation, the median score across all cells is shown. The values are relative to the score of cells that express gRNA-NT. **b** Values of the combined protein score that is shown in (**a**), ranked in descending order. gRNA*-Cebpb* gRNA*-Med12* targeted cells (green), gRNA-*Cebpb* or gRNA-*Med12*, (blue), gRNA-NT (red). **c** The expression score of a set of genes that regulate antigen presentation and T-cell co-stimulation. **d** Values of the score that is shown in (**c**), colored similarly to (**b**). **e** The expression of the CD86 protein, based on CD86 ADTs from the single-cell RNA-seq (scRNA-seq) data. Normalized UMI’s are scaled to gRNA. **f** FACS analysis showing the level of CD86 for CD11c-positive cells that were infected with lentivirus that encodes gRNAs as indicated. **g-h** ATAC-seq experiment in targeted BMDCs, technical repeats are shown. **g** Heatmap of genomic loci with significantly (abs(log2FC) > 1, false discovery rate (FDR) < 0.05) increased (red) or decreased (blue) chromatin accessibility. *P* values were calculated using two-tailed Wald test, and Benjamini-Hochberg was used to calculate the FDRs. **h** Differential accessibility (log2FC, *x* axis) and its significance (FDR, y axis) underlying significantly open or closed genomic regions in double knockout targeted cells. The dashed line corresponds to FDR = 0.00001. Each dot represents the peak with the lowest FDR for each gene. In purple genes with less accessible chromatin regions in targeted cells and in green genes with more accessible chromatin regions in targeted cells. Yellow dots mark significant genes with FDR > 0.00001. **i** Gene expression based on the high-order perturb-seq experiment. For each gene, UMIs were normalized to related expression in gRNA-NT cells.

We next leveraged the power of HMPCITE-seq to estimate the CD86, PD-L1, and MHC-I/CD45 protein levels in each combination of perturbations (Supplementary Fig. 11d–f). In addition, we computed a protein score by adding the normalized protein levels of CD86 and MHC-I/CD45, and subtracting the normalized protein level of PD-L1 (Fig. 5a, b, “Methods”). The combination of gRNA- *Prkar1a* gRNA-*March1* had the highest score, followed by gRNA-*Med12* gRNA-*Chd4*, gRNA-*Med12* gRNA-*March1, and* gRNA-*Med12* gRNA-*Cebpb*.

As a second criterion for the comparison between different combinations of targeted genes, we computed a signature score based on the genes that are involved in antigen processing and presentation and participate in T-cell co-stimulation (based on GO: 0019882, GO: 0031295, “Methods”). gRNA-*Med12* gRNA-*Cebpb* expressing cells had a high score also based on this criterion (Fig. 5c, d), with a strong elevation in genes that are involved in antigen processing, such as *Tap1/2*, *Clec4a*, and *Cd1d1* that mediate lipid presentation (Supplementary Fig. 11g–i). In addition, we could detect a clear induction of CD86 levels in the double perturbed *Cebpb* and *Med12* cells compared to cells that expressed only one of the gRNAs, according to both the FACS analysis and the scRNA-seq data (Fig. 5e, f).

Thus, based on the protein co-stimulatory score, the T-cell activation score, and CD86 expression, combined targeting of *Med12* and *Cebpb* is predicted to induce stronger DC activation and enhance T-cell priming.

To achieve a better understanding of MED12’s role, we targeted *Med12* in BMDCs and human monocytes (Supplementary Fig. 12). Key DEGs had an opposite trend compared to *Cebpb* perturbation in the BMDCs and human differentiated monocytes. For example, gRNA-*Med12* targeted cells showed reduced expression of receptors that mediate cell migration, as well as reduced, *Nr4a3* expression, but the expression of *Tnfrsf9* (4-1BB) which mediates T-cells priming was elevated (Supplementary Data 18 and 19).

Importantly, an ATAC-seq experiment using gRNA-*Med12* gRNA-*Cebpb-*targeted BMDCs showed enhanced chromatin accessibility at the *Tnfsr9* and *Tnfs9* but also of *Nr4a3 *loci (Fig. 5g, h), and increased expression according to the perturb-seq experiment results (Fig. 5i). Thus, targeting both *Cebpb* and *Med12* in the same cells have complementary effects on key genes that may improve BMDC activity and T-cell priming.

**Fig. 6:**
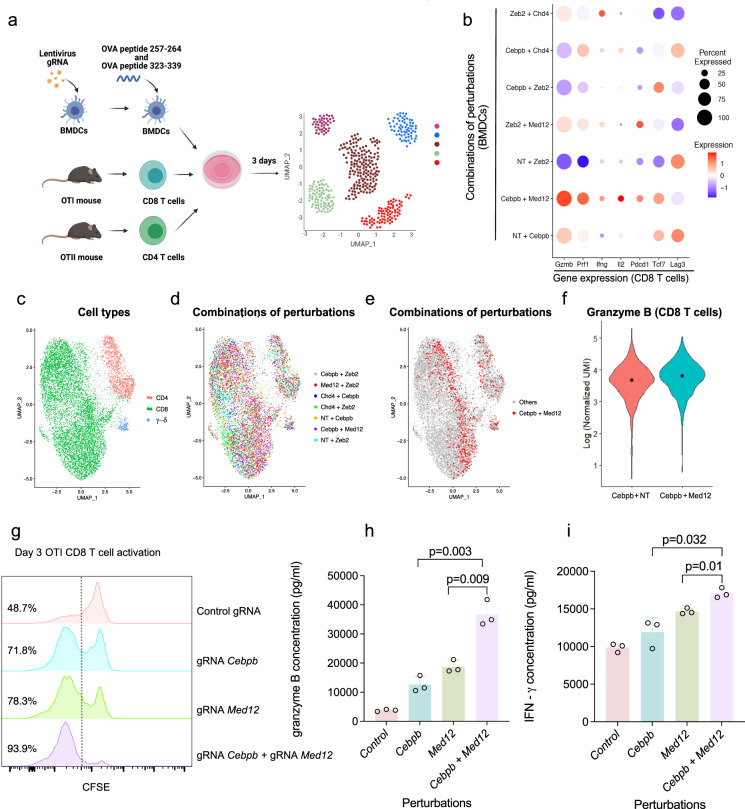
Targeting *Med12* and *Cebpb* in BMDCs enhances T-cell proliferation and cytokines secretion. **a** Cartoon showing the experimental setting. Perturbed BMDCs were co-incubated with OT-II CD4 and OT-I CD8 T cells for three days and then stained with hashing antibodies before single-cell RNA sequencing. 8885 cells are shown. **b** The abundance and average expression of differentially expressed genes across CD8 T cells from different samples. Dots are scaled across genes. **c** Uniform manifold approximation and projection (UMAP) representation of single-cell transcriptomes after removal of myeloid cells from the analysis. CD4, CD8, and gamma-delta T cells are shown. **d** The distribution of T cells across different samples. The targeted genes in BMDCs are shown in the legend. **e** T cells that were incubated with BMDCs that express gRNA*-Cebpb* gRNA-*Med12* are colored in the UMAP. **f** The distribution of *Gzmb* expression in OT-I CD8 T cells that were incubated with gRNA*-Cebpb* or gRNA*-Cebpb* gRNA-*Med12* (LFC = 0.17, FDR = 0.016, Wilcoxon Rank-Sum test). **g** Measure of CD8 T-cell proliferation after co-incubation with perturbed BMDCs. Three experiments were performed (*n* = 3), and a representative experiment is shown. **h**, **i** Results of ELISA for granzyme B and interferon-gamma following incubation of perturbed BMDCs and OT-I CD8 T cells (two-tailed unpaired *t*-test. *n* = 3 biologically independent samples). Data are presented as mean values ± SD. Source data are provided as a Source Data file.

To examine the actual effect of *Cebpb Med12* perturbations, we co-cultured targeted BMDCs that were incubated with OVA peptides, together with OT-I and OT-II T cells (Fig. 6a). We performed scRNA-seq experiment to profile the T cells and found that CD8 T cells were clustered according to the combinations of perturbations in BMDCs (Fig. 6c–e, Supplementary Fig. 11j, k). CD8 T cells that were co-cultured with gRNA-*Med12* gRNA-*Cebpb* BMDCs expressed higher levels of *Gzmb*, interferon-gamma and perforin and a lower level of *Lag3* compared to the single *Cebpb-*targeted cells and compared to other four combinations of perturbations that we tested (Fig. 6b, f). We also tested the effect of gRNA-*Med12* gRNA-*Cebpb* BMDCs on CD8 T-cell priming and found that the double KO cells better induced CD8 T-cell proliferation and enhanced secretion of *Gzmb* and interferon-gamma compared to the single KOs (Fig. 6g–i).

Finally, we examined the effect of perturbed BMDCs on immune cells that infiltrate tumors. We followed the procedure described in Fig. 2c, injecting gRNA-NT, gRNA-*Med12*, gRNA-*Cebpb, or* gRNA-*Med12* gRNA-*Cebpb* BMDCs to B16 tumor-bearing mice and performed scRNA-seq of the dissected tumors (Supplementary Fig. 13, Supplementary Data 20). We detected an increase in CD8 T cells (cluster 0) and a decrease in CD206 (*Mrc1*) positive macrophages (cluster 2) in tumors that were injected with gRNA-*Med12*. A similar trend was also seen in gRNA-*Med12* gRNA-*Cebpb* BMDCs injected tumors. This preliminary result is consistent with the enhanced expression of *Cxcl10* by *Med12* targeted BMDCs (Fig. 4k). However, further investigation of the functional effect of gRNA-*Med12* gRNA-*Cebpb* DCs on CD8 T cells killing and tumor growth is still needed.

Together, these results show that a combination of perturbations of *Med12* and *Cebpb* in antigen-presenting cells enhances the activation program, restricts the suppressive program, and most significantly, improves the proliferation and secretion of cytokines that are important for CD8 T cells function.

## Discussion

Most immunotherapy-based treatments aim to directly modify T cells. However, enhancing T-cell priming by dendritic cells, which stimulates the immune activation in vivo in response to infections or cancer, may create a more natural response in a manner that will not depend on a low number of antigens, thus establishing the correct balance between effector and memory T cells. Previous studies demonstrated that genetic modifications or an increased number of DCs in vivo, enhance an immune response and restrict tumor growth, even in cancer types that are resistant to immunotherapy-based treatments^17,18,52,53^.

Here, we screened for genes that are capable of changing the balance between the expression of co-stimulatory and co-inhibitory receptors, CD86 and PD-L1, that are expressed by DCs and play a critical role in regulating T cell priming and activation. We found and validated more than ten new targets that control the expression of these receptors. It will be interesting to examine whether PD-L1 regulators in innate immune cells also mediate the expression of PD-L1 in other immune cells and in cancer cells.

We have shown that overexpression of CD86 enhances T-cell proliferation, thereby demonstrating that CD86 expression is a rate-limiting factor in T-cell priming. Importantly, we showed that targeting top-ranked validated hits of the screens has a broader effect on the transcriptome, increasing the expression of genes that are involved in antigen processing and presentation, such as *Tap1* and *Tap2*, and pivotal receptors and cytokines such as MHC-I/II molecules, IL-12, and TNF alpha, that can further enhance the adaptive immune response. Thus, the results of our approach are not limited to the selection of the screen readout.

The finding of 13 subunits of the mediator complex that regulates transcription initiation in eukaryotes as negative regulators of CD86 is of special interest. We could not observe any reduction in cell viability, thus it is important to further investigate how this complex regulates the activation of APCs.

Knocking out *Cebpb* affects the cell state and increases the expression of a set of genes including *Ccr7* and *Fscn1*. These genes mark a subtype of DCs, mregDCs /PD-L2 positive DCs^41,43^, that were shown to infiltrate tumors. Importantly, it was shown that the differentiation of these cells from BMDCs depends on IRF4, and we detected a strong elevation of IRF4 levels in *Cebpb*-targeted cells. MregDCs (mature DCs enriched in immune regulatory molecules) cells were not predicted to have an anti-tumor effect based on previous publications^41^. However, the reduction in tumor growth after transferring *Cebpb*-targeted cells (Fig. 2), suggests that this DC subset can be utilized to restrict tumor growth. Therefore, targeting *Cebpb* provides an opportunity to produce a large number of DCs of this subtype. Importantly, mature DCs are characterized by a high expression level of co-stimulatory molecules and MHC complexes, together with a reduction in phagocytosis ability and antigen processing. Our in vitro and in vivo experiments (Fig. 2 and Supplementary Fig. 2) indicate that *Cebpb*-targeted cells can function in vivo as they capture and process antigens, activate T cells, and restrict tumor growth. These findings exemplify the therapeutic potential advantage of using genetic manipulations of DCs to improve their function, in comparison to using interferon or TLR agonists for DC maturation. Notably, targeting *Cebpb* increases the co-stimulatory and migratory programs in human mo-DCs as well (Supplementary Fig. 8). Together, these results emphasize the translational potential of generating mregDCs/PD-L2 positive cells by targeting *Cebpb*.

Genome-wide CRISPR screen results produce many target genes that can improve a desired cellular function but it remains challenging to determine which of these genes should be targeted based on the screening experiment. CRISPR screens followed by a perturb-seq experiment were previously done to find the effect of targeted hits on gene expression for improving T-cell proliferation and function^54–56^. However, previous works did not take advantage of high-order perturb-seq experiments in finding combinations of genes to target. High-order perturb-seq experiments with a set of genes that have a similar effect on the CRISPR screen readout revealed: (i) the effect of each perturbation on the expression profile, (ii) the genetic interactions between the genes that were validated in the genome-wide CRISPR screens, and (iii) the effect of combinations of targeted genes on the transcriptome, protein expression, and cellular function. By targeting 11 genes, we found that each perturbation had a different effect on gene expression, including a different effect on cytokines and cell surface receptors that are expressed by DCs and control the induction of the adaptive immune response. Targeting both *Cebpb* and *Med12* simultaneously increased the expression of CD86, and enhanced the expression of genes that are involved in antigen processing and co-stimulation of T cells, compared to single knockouts or other perturbation combinations. Consistent with these findings, CD8 T cells that were incubated with gRNA-*Cebpb* gRNA-*Med12* double KO BMDCs, had higher expression of interferon, *Gzmb*, and perforin, and low expression of *Lag3*. These alterations in gene expression and cytokine secretion (Figs. 5 and 6) suggest enhanced cancer cell-killing potential, although we did not assess cancer cell-killing directly. We experienced technical challenges in generating a large number of immune cells that were perturbed in more than one gene. This technical obstacle needs to be solved to apply our methodology in vivo or in a clinical setting. In addition, it is important to note that cancer cell killing by T cells in vivo is affected by many environmental signals including suppressive cytokines, nutrient availability, and different obstacles, such as desmoplastic barriers. Such immunosuppressive signals can differ between cancer types and it may be worth applying HMPCITE-seq in the presence of relevant immunosuppressive conditions.

In conclusion, HMPCITE-seq can be utilized to improve immune cell function and assist in choosing a combination of genes to target in various scenarios.

## Methods

### Ethical statement

This research complies with all relevant ethical regulations. All experiments involving Human tissue were carried out in accordance with ethical guidelines and were authorized by the Hadassah Medical Center Committee for Human Experiments. All procedures involving animals were done in compliance with the National Institutes of Health and Institutional guidelines with approval from the Institutional Animal Care and Use Committee of the Hebrew University (Jerusalem, Israel). The maximal tumor size permitted by the committee is 1 cm, which was not exceeded in the in vivo procedures.

### Human samples

Human peripheral blood mononuclear cells (PBMCs) were obtained from the Hebrew University-Hadassah Medical Center (Jerusalem, Israel). Informed consent was obtained from all the participants and the studies were conducted in accordance with ethical guidelines (0268-19-HMO Declaration of Helsinki) and were approved by the Hadassah Medical Center Committee for Human Experiments (Helsinki Committee).

### Mice

All animal protocols were approved by the Joint Ethics Committee (Institutional Animal Care and Use Committee) of the Hebrew University (Jerusalem, Israel) and Hadassah Medical Center (Jerusalem, Israel). The ethics protocol for mice holding and the experimental procedures are MD-18-15411-5 and MD-19-16071-1. The following mice strains were purchased from the Jackson Laboratory: C57BL/6-Tg (*Tcra Tcrb*) 1100Mjb/J (stock #003831), B6.Cg-Tg (*Tcra Tcrb*) 425Cbn/J (stock# 004194). Cas9-expressing mice (stock #026556), BALB/cJ-*Cebpb* tm1.1Elgaz/J (stock #032282), C57BL/6J-Tg (*Itgax-cre-EGFP*) 4097Ach/J (stock #:007567). *Rag*^−*/*−^ mice were obtained from Prof. Yinon Ben Neriah. The C57BL/6 mice (five weeks) were purchased from Envigo Israel.

### Cell lines

B16-F10 (ATCC CRL-6475) cells and B16-OVA cells (kindly provided by Prof. Michal Lotem) were maintained in DMEM medium (Gibco, #41965039) supplemented with 10% FCS and 1% sodium pyruvate (Gibco, #11360039). 293T cells (ATCC CRL-3216) were maintained in DMEM medium (Gibco, #41965039) supplemented with 10% FCS and 1% sodium pyruvate (Gibco, #11360039).

### Differentiation of bone marrow-derived dendritic cells (BMDCs)

To obtain BMDCs, femurs and tibia were removed from eight-week-old mice and the bone marrow was flushed. Red blood cells were lysed using RBC lysis buffer (e-bioscience). Bone marrow cells were plated in 100 mm diameter dishes (Falcon, BD, NJ, USA) at a concentration of 2 × 10^5^ per ml in 10 ml of medium. The medium included RPMI 1640 (Gibco, #21875-034) supplemented with 10% heat-inactivated FBS (Gibco, #10270106), β-mercaptoethanol (50 µM, Gibco, #31350-010),1% l-glutamine (Gibco, #25030-024), 1% penicillin/streptomycin (Gibco, #15140-122), 1% MEM non-essential amino acids (Gibco, #11140035), 1% HEPES (Gibco, #15630056), 1% sodium pyruvate (Gibco, #11360-039), and murine GM-CSF (20 ng/ml; Peprotech, #315-03). For CRISPR knockout experiments 6- to 8-week-old constitutive Cas9-expressing mice were used as the source of BMDCs. Cells were maintained at 37 °C in a 5% CO_2_-humidified atmosphere. On day two of the culture, 10 ml of fresh medium supplemented with GM-SCF was added. On day five, 10 ml of medium was gently removed and 10 ml of fresh medium with GM-CSF was added. On day seven, 5 ml of fresh medium with GM-CSF was added to the cells and on day eight, floating cells were recovered and used for downstream applications.

### Antibodies and drugs

The following fluorescent antibodies were used in this study in a 1:200 dilution ratio:

Screens: CD11c (BLG-117310), CD274 (BLG-124307) and CD86 (BLG-159203).

Single gRNA validations: CD11c (BLG-117322), CD274 (BLG-124307), CD86 (BLG-105012).

Additional markers: CD80 (BLG-104708), MHC-I (H-2Kb) (BLG-116505), CCR7 (CD197) (BLG-120107), MHC-II I-A/I-E (BLG-107606), IL-12/IL-23 p40 (BLG-505204) and TNF-α (BLG-506306).

Hashtag-oligos (HTO) and antibody-derived tags (ADT): TotalSeqTM-A0301 (BLG-155801), TotalSeqTM-A0303 (BLG-155805), TotalSeqTM-A0304 (BLG-155807), TotalSeq™-A0306 (BLG-155811) TotalSeq™-A0309 (BLG-155817). TotalSeqTM-A0200 CD86 (BLG-105047), TotalSeqTM-A0190 CD274 (BLG-153604).

For the human monocytes experiments, the following antibodies were used in a 1:50 dilution ratio: CD11c (BLG-301623), CD14 (BLG-367115), PD-L1 (BLG-329706), CD86 (BLG-374207), CD1c (BLG-331523), CD209 (BLG-330107), CD11b (BLG-101205), CD141 (BLG-344103).

Myriocin, 10 nM of Myriocin (Sigma-Aldrich), was added to BMDCs on day seven and Flow cytometry (FACS) analysis of PD-L1 expression was performed on day eight.

The following reagents were added on day eight to cultured BMDCs for 12 h: LPS (0.1 ng/ml, InvivoGen, # 14D09-MM), 5 ng IFN beta (3.3 ng/ml, R&D systems, #8234-MB-010) or Poly I:C (25 µg/ml, Sigma, # P9582).

### Lentiviral production and transduction

Lentivirus was produced by triple transfection of HEK-293 T cells with a lentiviral transfer vector and the packaging plasmids psPAX2 vector (Addgene. #12260, psPAX2 was a gift from Didier Trono http://n2t.net/addgene:12260; Addgene, #12260) and pVSVg (Addgene #31947^57^) at a 4:3:1 ratio. Transfection was performed using PEI max transfection grade (Polysciences, Inc #24765) in a stock concentration of 3 µg/µl and OptiMEM (Gibco, #31985047). For each reaction, 0.7 µl of PEI was used for 1 µg of DNA. The next morning, the medium was removed and replaced with fresh BMDC media as previously described. The supernatant was collected 48 and 72 hours after transfection, filtered through a 0.45 μm filter, and added to the cells.

To produce lentivirus for the screens, viral particles were concentrated using Corning® 50 mL centrifuge tubes (Sigma) for 3 hours at 4 °C (119802 RFC). The virus was aliquoted and frozen at −80 °C. The titer of the virus was measured by using BMDCs from C57BL/6 mice followed by puromycin selection.

### Genome-wide CRISPR screens

To perform genetic screens, we followed the BMDCs differentiation protocol described above with several modifications. First, 200 × 10^6^ cells were collected from constitutive Cas9-expressing females. On day two, cells were infected with the pooled lentiviral library at an MOI of one and eight hours later fed with additional media supplemented with GM-CSF as described above. On day eight, all non-adherent and loosely adherent cells were collected and harvested by centrifugation. BMDCs were washed twice with PBS and stained with CD11c, and CD86 or PD-L1 antibodies. CD11c-positive cells were gated and sorted into two bins, 10% high and low expression of CD86 or PD-L1 using a BD FACSAria™ III sorter. Two repeats of the genome-wide CRISPR screen were performed using CD86 as a readout and one genome-wide CRISPR screen was done using PD-L1 as a readout. The Brie pooled gRNA library (including non-targeting sgRNA) was used in the genome-wide screens^26^. Custom libraries for the secondary screens, which included gRNA for 2300 genes and non-targeting sgRNA were designed based on a previous study^58^.

### Custom library cloning

Top-ranked positive and negative regulatory genes were chosen for the secondary screens. An oligo pool was ordered from Twist Bioscience and was amplified using the following primers:

Forward: TAACTTGAAAGTATTTCGATTTCTTGGCTTTATATATCTTGTGGAAAGGACGAAACACCG

Reverse: ACTTTTTCAAGTTGATAACGGACTAGCCTTATTTTAACTTGCTATTTCTAGCTCTAAAA

The amplified DNA was cloned into a lentiGuide-Puro vector (Addgene #52963) for the PDL-1 screen or into CRISPseq-BFP-backbone (Addgene #85707) for the CD86 screen at 1:3 vector to insert ratio using the Gibson Assembly Mix (NEB Builder HiFi DNA Assembly #M5520A). The final ligation reaction was diluted at 1:2 with molecular-grade water and used for the transformation of electrocompetent bacteria (Enduro DUOs cells, Lucigen, #60242-2). The transformation was conducted with Gene Pulser Xcell (BioRad). gRNA coverage of x30 was obtained. Plasmid DNA was extracted with NucleoBond Xtra Midi Kit (#MAN-740410.50). Two repeats of the secondary screen using PD-L1 as a readout, and one repeat using CD86 as a readout were executed.

### DNA purification and gRNA sequencing

DNA was purified using Qiagen DNeasy Blood & Tissue Kit (Qiagen #69504) according to the manufacturer’s instructions and eluted in 50 µl H_2_O. We performed two successive PCR reactions described previously using GoTaq-Green Master Mix (Promega Cooperation #M7122). Five microliters from the first PCR were used for the second PCR (50 µl) with primers that include the Illumina adapters^5^. The final PCR product was purified from a gel using QIAquick Gel Extraction Kit (Qiagen #28704). Libraries were sequenced using a Nextseq 500 machine (Illumina).

### Cloning and design of gRNAs

To design single gRNAs, we either used the sequences from the secondary libraries (Supplementary Data 2 and Supplementary Data 4) or used the CrispRGold design tool^59^ (Supplementary Data 21).

For individual gRNA cloning, pairs of oligonucleotides (IDT) with *BsmBI*-compatible overhangs were annealed and cloned into the CropSeq-Guide-Puro plasmid (Addgene, plasmid #86708). Five micrograms of plasmid, 2 µL *BsmBI* enzyme (NEB #R0580), 1×NEB 3.1 adjusted with DDW to a 40 µl volume reaction were incubated for two hours at 55 °C and then incubated for an additional 30 min at 37 °C with 2U *shrimp alkaline phosphatase* (rSAP, NEB #M0371S). The vector was then cleaned on a PCR purification column (Qiagen #28704) to remove enzymes, salts, and small DNA fragments.

The insert was prepared as follows:

N(20) was the guide target sequence, and n(20) was the reverse complement:

Forward: 5′ CACCG NNNNNNNNNNNNNNNNNNNN 3′

Reverse: 5′ AAAC nnnnnnnnnnnnnnnnnnnn C 3′

Sense and antisense oligos were mixed at a final concentration of 50 µM each in DDW. The reaction was set as follows: 1 µL oligo mix, 1 µL T4 ligase buffer (10x) (NEB #B0202S), 0.5 µL *T4 PNK* (NEB #M0201S), and 7.5 µL water. Oligos were phosphorylated and annealed in a thermocycler and diluted 1:200 in DDW after completion of the reaction.

For ligation, 1.5 µL of the annealed and diluted oligos were used together with 1 µL from a 20 ng/µL concentration of the *BsmBI*-digested lentivector, 0.5 µL T4 ligase buffer (10×) (NEB #B0202S), 1.9 µL DDW, and 0.1 µL *T4 ligase* (NEB #M0202L) per one reaction. The reaction was incubated at 16 °C overnight.

The transformation was performed using 20 µL of chemo-competent cells and 5 ng of the ligation mixture. The reaction was incubated on ice for 30 min, and then heat shocked for 30 s at 42 °C. Cells were then recovered for 1 h using LB media at 37 °C while being shaken at 180 RPM. Following, 100 µL of the cells were spread on ampicillin-agar plates and incubated overnight at 37 °C. To determine the insertion of the guides, single clones were sent for Sanger sequencing after mini-prep (HiYield™ Plasmid Mini Kit-300 #YPD300).

The mCherry vector that was used in this study was generated from LentiCRISPRv2-mCherry vector (addgene #99154). The Cas9 sequence was removed using AfeI (NEB #R0652S) and BamHI (NEB #R0136S). A blunt end was created using the Klenow Fragment (ThermoFisher #EP0051) followed by ligation overnight using *T4 DNA Ligase* (NEB #M0202L).

A CD86 overexpression vector was cloned into an N174-MCS (Puro) vector (Addgene #81068). Cd86 cDNA was amplified from BMDCs with a pair of primers that contains Not1 and Ecor1 restriction enzyme cutting sites and flanking bases. After that, the N174-MCS (Puro) vector and the cDNA of Cd86 were digested by *NotI* (NEB #R3189L) and *EcoRI* (NEB #R3101L) restriction enzymes for 4 h, and the fragment was ligated overnight using *T4 DNA ligase* (NEB #M0202L). Primers for Cd86 cDNA amplification: Forward: CGGAATTC GAACTTACGGAAGCACCCAC

Reverse: ATTTGCGGCCGCCTCTTTCCTCAGGCTCTCAC

For the in vivo B16 scRNA-seq experiment, we cloned two gRNA cassettes in the same lentivector. To clone the second cassette, we PCRed a vector that includes the desired gRNA with 10 µM. Forward primer:

GAGCAAAACAAAAGTAAGACCACCGCACAGCAAGCCATATGGAGGGCCTATTTCCCATGATT

Reverse primer:

CCCTCATATCTCCTCCTCCAGGTCTGAAGATCAGCCTCAAGATCTAGTTACGCCAA

We then digested the Addgene plasmid #52963 with *NotI* enzyme (NEB # R3189L) and used Gibson Assembly Mix (NEB Builder HiFi DNA Assembly #M5520A) for cloning.

### Tumor induction and injection of BMDCs

A total of 5 × 10^5^ B16 cells were injected subcutaneously into C57BL/6 or Rag1^−/−^ C57BL/6 recipient mice. Five days later, a total of 5 × 10^6^ lentiviral infected BMDCs were injected subcutaneously into tumor-bearing mice. The size of the tumors was measured using the formula *V* = (*W*^2^ × *L*)/2 and the mice were sacrificed when the tumor diameter reached 1 cm. The size and weight of tumors were measured at the end of the experiment. Alternatively, tumors were digested for scRNA-seq experiments.

### B16 tumor digestion

Tumors were minced and digested in DMEM media supplemented with 2.5 mg/ml Collagenase D (Roche, #11088866001) and 0.2 mg/ml *DNaseI* (Roche, # 10104159001). After incubation and shaking in the water bath at 37 °C for 10 min, samples were strained through 100 mm filters and centrifuged at 1500 RCF at 4 °C for 5 min. Cell pellets were resuspended in 1 ml of ACK lysis buffer (Gibco, #A100492-01), incubated for 5 min at RT, and washed twice with quenching buffer (PBS + 2% FCS).

### Spleen digestion

Spleens were cut with scissors into fragments less than 1 mm and mixed with digestion buffer consisting of RPMI media supplemented with 2% FCS, 1 mg/ml Collagenase D (Roche, #11088866001) and 20 μg/ml DNaseI (Roche, # 10104159001). Samples were incubated in the water bath at 37 °C while shaking for 30 min. After digestion, samples were strained through 70-mm filters and washed in serum-free RPMI media. Red blood cells were lysed using RBC lysis buffer (Sigma, #R7757).

### Ex vivo T-cell proliferation assay

Mice were injected with 5 × 10^5^ B16 or B16-OVA cells. After 5 days, BMDCs which were labeled with a CFSE cell division tracker kit (BioLegend #423801) were injected subcutaneously on their sixth day of differentiation. Two days later, FITC-positive cells were sorted from inguinal lymph node and co-cultured with CFSE-labeled OT-I CD8+ T cells in a 1:1 ratio for 3 days. The proliferation rate of CD8+ T cells was evaluated by FACS.

### Cytokine production assay

Modified and sorted BMDCs and OT-I CD8+ T cells were co-cultured together at a 1:5 ratio in a flat-bottom 96-well plate for 3 days. Culture supernatants were collected and analyzed using an ELISA assay. IFN-γ and granzyme B were detected with the ELISA kit (Invitrogen # 88-7314, Invitrogen #88-8022) using the manufacturer’s instruction manual.

### In vitro T-cell proliferation assay

BMDCs were pretreated with OVA (257–264) SIINFEKL (InvivoGen #vac-sin) for 4 h or pretreated with OVA protein (InvivoGen #vac-pova) overnight. OT-I CD8+ T cells were isolated from the spleen of OT-I mice using EasySep™ Mouse CD8+ T-Cell Isolation Kit (STEMCELL Technologies #19853). The CD8+ T cells were labeled using the CFSE cell division tracker kit (Biolegend #423801). The BMDCs and OT-I CD8+ T cells were co-cultured at a 1:4 ratio for 2 days in a flat-bottom 96-well plate (Corning #3596). In the experiments that are shown in Fig. 6, BMDCs were pulsed with OVA (257–264) SIINFEKL peptide (InvivoGen #vac-sin) and OVA peptide (323–339) (InvivoGen #vac-isq) overnight. OT-I CD8+ T cells and OT-II CD4+ T cells were isolated and stained with CFSE. The BMDCs and the mixture of OT-I CD8 positive T cells and OT-II CD4 positive T cells were co-cultured at a 1:4 ratio for 3 days in a flat-bottom 24-well plate (Corning #3596).

### Cell migration assay

Cell migration assays were performed using an 8.0 µm pore polycarbonate membrane insert plate Transwell® (Corning, Life Science #3422). BMDCs were added to the upper chambers and the ligands CCL19 (R&D System #440-M3-025) and CCL21 (R&D System #457-6C-025) were added to the lower chambers at a 50 ng/μL concentration. Eight hours later the number of migrated DCs in the lower chamber was evaluated by FACS.

### Phagocytosis assay

The phagocytosis assay was performed using a phagocytosis assay kit (Abcam, #ab234054). BMDCs were infected with BFP-gRNA-*Cebpb* or BFP-gRNA-non-targeting virus. The control cells were pretreated with 5 μg/ml Cytochalasin D (Sigma, #C2618-200UL) for 1 h. All cells were treated with 5 μL of Zymosan slurry for 2 h. Cells were washed accordingly and Zymosan-positive cells were evaluated by FACS.

### Bulk RNA sequencing

RNA was recovered using an RNeasy plus mini kit (Qiagen #74136). The libraries were prepared using a KAPA mRNA capture kit (Roche #07962231001) following the manufacturer’s instructions.

### Perturb-seq experiments

The gRNAs were cloned into CROPseq-Guide-Puro (Addgene, #86708)^44^. All the gRNAs were pooled together in equal concentrations. BMDCs were transduced and selected with puromycin in the experiments that are shown in Supplementary Fig. 5 and Fig. 4. In the experiment in Fig. 3, the cells were not selected.

For the experiment shown in Supplementary Fig. 5, we used gRNA-*Cd86*, gRNA-*CD274*, and gRNA-non-targeting. On day 8, cells were incubated with CD86 and PD-L1 barcoded antibodies and washed three times with cold PBS.

For the double targeting *Cebpb* and *Nr4a3* experiment in Fig. 3, gRNA-*Cebpb* and gRNA-*Nr4a3* were cloned into the CROPseq-Guide-Puro (Addgene #86708).

For the experiment in Fig. 4, a pool of 32 gRNAs was cloned into CROPseq-Guide-Puro (Addgene #86708). HashTag Oligonucleotide (HTO) antibodies and anti-CD86 and anti-PD-L1 ADT TotalSeq™ antibodies were used. After three washes with cold PBS, 50,000 cells were loaded on seven channels of 10X Genomics Chromium single-cell 3′ chip.

HTO and ADT sequences were recovered from the cDNA using the manufacturer protocol^12^. gRNA sequences were recovered from the cDNA libraries.

CDNA, ADT, HTO, and the gRNA libraries were sequenced using Novaseq6000 (Illumina).

### Recovery of human monocyte and nucleofection with Cas9-ribonucleoprotein complex (RNP)

Peripheral blood mononuclear cells (PBMCs) were isolated using a density gradient (Lymphoprep, STEMCELL Technologies, #7851) as previously described^60^. One buffy coat from Hadassah Medical Hospital was processed for each donor using BSL2* precautions in accordance with institutional safety guidelines. The buffy coat was diluted 1:1 with PBS (BI 02-023-1A), then layered on top of the lymphoprep in a 50 ml tube (Lifegene, LBT50). Isolated PMBCs were then cryopreserved in a mixture containing FBS with 10% DMSO (40 × 10^6^ cells in 1 ml) and stored in liquid nitrogen, or used directly for monocyte isolation. Monocytes were isolated using the EasySep Human Monocytes Isolation Kit (STEMCELL Technologies, #19359) according to the manufacturer’s instructions. To examine isolation efficiency, fractions of cells before and after separation were stained with human anti-CD14 antibody and analyzed using flow cytometry.

Directly after isolation cells were nuclefied for downstream applications.

Human monocytes were cultured in 1XIMDM media (Gibco, #12440053) supplemented with 1% FBS (Rhenium #10270106), penicillin–streptomycin (100 IU and 100 ug/ml), 1 mM sodium pyruvate, 50 ng/ml human GM-CSF (Peprotech, #300035) and 50 ng/ml human IL-4 (Peprotech, #0200-04-05).

To perturb the monocytes, isolated cells were spun down for five min at 300 × *g* and washed twice with OptiMEM (Gibco, #31985047). Monocytes were resuspended at a concentration of 1 × 10^6^ cells per 94 μl of OptiMEM. Cas9-ribonucleoprotein (RNP) complex formation was done according to the protocol described by Hendel et al. (User method: Electroporation of primary human CD34+ hematopoietic stem and progenitor cells)^17–19^. Briefly, for each electroporation reaction, the following mixture was prepared by gentle pipetting: 2.1 μl of PBS, 1.2 μl (120 pmol) of Alt-R gRNA (designed by IDT, www.idtdna.com/CRISPR-Cas9), 1.7 μl (105 pmol, 62 μM) of Alt-R S.p. Cas9 Nuclease V3 (IDT #1081058). The RNP complex was incubated at room temperature for 15 min and combined with 1 μl (3.85 μM) of electroporation enhancer (IDT #1075915). Then, 94 μl of cell suspension were added to the RNP and transferred into the cuvette (2 mm gap) for further nucleofection. Nucleofection was done using NEPA 21 machine (NEPA GENE) according to the instruction manual with the settings described in Supplementary Table 1. Immediately after nucleofection, 400 μl of pre-warmed media supplemented with 50 ng/ml of human GM-CSF (Peprotech, #300035) and 50 ng/ml of human IL-4 (Peprotech, #0200-04-05) were added to the cuvette, and the whole volume was transferred and split into two wells of a 48 well plate (Tamar, #83.3923.500). After nucleofection, cells were fed as previously described^6^. Briefly, 24 hours post nucleofection, the entire volume of media was removed and substituted with the fresh media. On day 3 and day 5 post nucleofection, half of the volume of the media was removed and an equivalent volume of the fresh media was added.

Alt-R gRNAs were synthesized by IDT and resuspended in 1× Nuclease-free IDTE, pH 7.5 (1× TE solution) (IDT #11-01-02-02) at a concentration 100 μM. The sgRNA sequences are indicated in Supplementary Table 1.

### Quantitative PCR

RNA was recovered using an RNeasy Plus Mini Kit (Qiagen #74136). cDNA was generated using qScript cDNA Synthesis Kit (QuantaBio #95047-200). The qPCR reactions were prepared using PerfeCTa SYBR Green FastMix (Quantabio # 95074-012). Nr4a3 forward primer: GATCCACCTGCCCTGTAGAA, Nr4a3 reverse primer: ATTGGGCTTCTGAGTGGATG. Actb forward primer: CGCCACCAGTTCGCCATGGA, Actb reverse primer: TACAGCCCGGGGAGCATCGT.

### ChIP-seq

ChIP-seq was performed according to the protocol described by Antman et al.^61^ with several modifications. We used 3 × 10^7^ BMDCs for each experiment. Quenching of formaldehyde was done at RT on a gently rocking platform. Samples were sonicated for three cycles (30 s on, 30 s off). For immunoprecipitation, 10 µL of Cebpb antibody was added (ab32358) along with 30 µL of Protein G magnetic beads. Then, 2.5% of the sample was taken as input. Chromatin was eluted from the beads by shaking them in a heat block at 800 RPM for 30 min at 65 °C and reverse crosslinking was performed at 65 °C overnight. Next, 1 ng of immunoprecipitated sample and 100 ng of input sample were used for library preparation with KAPA HyperPrep Kit (Roche #7962347001). The library was sequenced using Novaseq6000 (Illumina).

### ATAC-seq

BMDCs were isolated, differentiated, and infected with lentivirus as described above. Cells carrying the corresponding KOs were sorted using BD FACSAria™ III sorter. Around 10^5^ cells were used per sample. Sample preparation for the ATAC-seq was done using the kit (Active Motif #53150) according to the instruction manual.

### Gene annotation

To find the transcription factors from the ChIP-seq enrichment analysis in Fig. 3e, f, we used Enrichr^62,63^. For gene ontology enrichment analysis in the text of the results section and in Supplementary Data Fig. 1g and 8, we used the Gene Ontology enRIchment anaLysis and visuaLizAtion tool, Gorilla^64,65^.

### CRISPR Screens analysis

Fastq files were demultiplexed using *Bcl2fastq* (v2.20.0.422) and converted to fasta files using the *fastx* toolkit. Forward and reverse adapters were trimmed using *cutadapt* (v3.4)^66^ and aligned to the reference library by *bowtie 1*^67^*. MAGeCK*^68^ was used to score the gRNAs and rank the genes. To examine the robustness of the gene ranking algorithm, we applied additional computational methods to compute the secondary screen results of the CD86 experiment (shown in Supplementary Fig. 1), including casTLE^69^, PBNPA^70^, and ranking based on Z-score calculation^4^.

### Bulk RNA-seq analysis

For the experiment shown in Supplementary Fig. 12b, FASTQ files were trimmed to remove adapters and low-quality sequences using trim-galore (v0.6.7) and then aligned to the reference genome Hg38 using hisat2 (v2.2.1) with the --very-sensitive option. Transcripts were quantified with stringtie (v2.2.1).

For the RNA-seq analysis in Fig. 3c, Supplementary Fig. 8 and Supplementary Fig. 12a raw reads were quality-trimmed and the remaining adapter sequences were removed using cutadapt (v2.10). Processed reads were aligned to the mouse genome version GRCm38 or Hg38 with TopHat^71^ (v2.1.1). Quantification was done with htseq-count^17^ (v0.6.0) with strand information set to ‘reverse’.

DESeq2^72^ (v1.26.0) analysis for the identification of differentially expressed genes was performed and pair-wise comparisons were tested with default parameters, except not using the independent filtering algorithm. For Fig. 3, the significance threshold for differentially expressed (DE) genes was taken as padj < 0.1 (default) and a baseMean-dependent log2FoldChange threshold, requiring a baseMean above 5 and an absolute log2 fold change higher than 5/sqrt(baseMean) + 0.3. Normalized counts per sample of DE genes in at least one of the contrasts were pooled together and scaled (subtracting the mean signal and dividing by the standard deviation) per row. The scaled expression matrix was subjected to k-mean clustering and *k* = 4 solution was selected. Results were shown as a heatmap, where genes were sorted by cluster assignment and samples were sorted by treatment.

For Supplementary Fig. 8 and Supplementary Fig. 12, mouse to human homolog tables were downloaded from the MGI database^73^. The two DE gene lists of mouse and human RNA-sequencing experiments were merged by matching pairs for which 1:1 homology was identified. Significant DE genes were identified by padj < 0.05 and a LFC threshold that was determined based on the LFC distribution of all genes in each experiment (abs(LFC) > 0.3 or 0.5 in Supplementary Fig. 8, abs(LFC) > 0.7 in Supplementary Fig. 12). Only significant genes with a matching homolog which showed the same fold change trend (Up/Down regulation) in both mouse and human experiments were selected for inclusion in the heatmaps. Heatmaps show the scaled normalized expression values of the selected genes after hierarchical clustering of the rows (genes).

For the experiment shown in Supplementary Fig. 3b, raw reads were quality-trimmed with TrimGalore (v0.6.4)^74^. Processed reads were aligned to the mouse genome version GRCm38 (Ensembl release 89) with the *STAR*^75^ (v2.7.3) aligner and quantified using *Salmon* (v0.14.0)^76^. The standard DESeq2^19^ workflow with default parameters was used to identify DEG between both conditions. Normalized counts per sample of selected DEG were pooled together and Z-score standardized per row. Hierarchical clustering on both rows and columns was performed.

### Processing and analysis of ATAC-seq and ChIP-seq data

Paired-end FASTQ files were trimmed to remove adapters and low-quality sequences using trim-galore (v0.6.7) and then were aligned to the mm10 reference genome using hisat2 (v2.2.1)^77^. picard (v2.26.10) MarkDuplicates was used to mark and remove duplicates. Bigwig files were generated using deeptools (v3.5.1)^78^, normalizing by RPKM or BPM. Genomic tracks were visualized using the Integrative Genomics Viewer (IGV). Heatmaps were generated using deeptools (v3.5.1) and visualized in R using profileplyr (v1.14.1).

Peaks for each sample were assigned using macs (v2.2.7.1)^79^. A union peak set for all samples was constructed by merging the peaks into a set of high-confidence non-overlapping fixed-width (500 bp) peaks. Differential peaks were determined using DESeq2^72^. ChIPseeker (v1.34.1)^80^ was used for peak annotation. For each comparison, significant peaks were defined as those with a false discovery rate (FDR) lower than 0.05 and fold change thresholds.

For comparison between RNA-seq and ATAC-seq data, the R package disco (v0.6)^81^ was used.

### Motif analysis

Motifs enriched in ChIP data were analyzed using the HOMER^37^. ATAC-seq enriched motifs were analyzed with chromVAR (v1.20.2)^82^.

### Single-cell RNA-seq analysis

Chromium single-cell 3′ chip (10x Genomics) libraries were sequenced and the reads were mapped to *mm10* library (Ensembl v98), which was modified to include the sequence of the CROPseq vector^5^. The alignment was performed using *Cellranger*^20^ (v4.0.0). Pre-processing of each GEM was done separately to identify sequencing batch effects.

### Computational analysis of ADT, HTO, and GDO modalities

ADT, HTO, and GDO fastq files were quality filtered using *cutadapt* (v3.4), and R2 was trimmed to exactly 20 base pairs. To generate count matrices for these modalities, a modified version of *CITE-seq-count* (V1.4.3)^83^ was constructed and implemented (*Custom Cite-seq)*. *Custom Cite-seq* allowed us to construct CBC-UMI-Feature-Reads matrices that were used for downstream analysis. Using *Custom Cite-seq*, we filtered out cases in which a CBC-UMI had more than one identified feature with a significant amount of reads. GDO UMIs that had less than five reads were filtered out.

After the initial processing of ADT/HTO/GDO matrices, downstream analysis was done using *Seurat* R package. Cells with a high total GDO UMI count (top five percentiles) were filtered out, as well as cells with a low number of detected cDNA UMI (<5000), or a low number of identified genes (<200).

HTO UMI counts were normalized using the centered log-ratio transformation, with a margin=1 (to normalize across features), and cells were demultiplexed by *MULTIseqDemux* from the *Seurat* (v4.0.4) R package^84^. In the in vivo scRNA-seq of B16 tumors, the HTO assignment was performed by HTODemux from the Seurat R package after filtering out non-immune cells. Cells that were not confidently identified to express a single HTO barcode, were filtered out. ADT UMI counts were normalized by using relative counts across features for receptor score calculation. For visualization purposes, ADT UMI counts were normalized by using centered log-ratio transformation with a margin = 1.

gRNA was assigned to a cell if it passed two separate thresholds: (i) a minimum of five UMIs, (ii) the UMIs for this gRNA must constitute at least 20% of the total GDO UMIs for this cell. Cells without an identified gRNA were filtered out, and gRNA combinations with less than 10 cells (mainly triplets and higher-order gRNA combinations) were discarded.

### Assignment of cells to cell groups

The *Ucell*^85^ (v1.1) R package was used for the assignment of cells to cell groups, based on gene lists that were generated by selecting top DE genes between clusters in the experiment shown in Supplementary Fig. 5.

### Regression and UMAP generation

In all scRNA-seq experiments with BMDC cells and in the in vivo scRNA-seq of B16 tumors, cell cycle regression was performed by the *CellCycleScoring* method from the *Seurat* package. PCA was performed based on log-normalized RNA data, followed by UMAP dimensional reduction. In the experiment involving splenocytes, after the initial UMAP preparation, lymphocytes were filtered out. In the experiment with B16 tumor, all cells apart from CD45 positive cells were filtered out.

### Perturb-Seq analysis - additional analysis

We performed several additional processing steps to obtain a quantitative estimate of HTO, which is correlated with the quantity of MHC-I on the cell surface. First, the cells were divided into different groups based on their most abundant HTO feature. Next, a relative count normalization was performed for each group separately, and the normalized HTO feature was stored as HTO-Max. Following that, the groups were combined and a unified HTO-Max variable was created.

In Fig. 4h–j and Supplementary Fig. 10m–q, a chi-square test for the distribution of perturbations (singlets) across clusters was performed, and standardized residuals for each perturbation were calculated.

### Calculation of gene expression signature score

The score was quantified by the non-parametric *Ucell* algorithm, based on lists of genes associated with antigen presentation and T cells co-stimulation (GO:0019882 and GO:0031295).

### Receptor score calculation

ADT and HTO matrices were normalized by using relative counts with the same scale factor and were summed to obtain the combined score. For each cell, the combined score was calculated as ADT CD86 normalized UMIs + HTO normalized UMIs - ADT CD274 normalized UMIs.

### Additional statistical analysis and plot generation and software

A comparison of continuous variables between two groups was performed by using a two-tailed student’s t-test (Figs. 2 and 3 and Supplementary Figs. 2 and 3).

To assess whether the number of overlapping genes in Fig. 1g was higher than expected by chance, a hypergeometric test was performed, with the population size set as the total number of genes in the reference transcriptome. Similarly, a hypergeometric test was used for Supplementary Fig. 8e, f and Supplementary Fig. 12c, d, with the population size set at the total number of genes with 1:1 homology between the hMo and mouse experiments.

Figures were generated by the GraphPad Prism 9.0 software, and by R (version 4.0.2). The following packages for R were used for plot generation: *ggplot2*^86^, *Seurat, ComplexHeatmap*^87^, *pheatmap*^88^. FlowJo software version 10.1r1 was used for FACS analysis.

### Reporting summary

Further information on research design is available in the Nature Portfolio Reporting Summary linked to this article.

### Supplementary information

Supplementary Information

Peer Review File

Description of Additional Supplementary Files

Supplementary Data 1

Supplementary Data 2

Supplementary Data 3

Supplementary Data 4

Supplementary Data 5

Supplementary Data 6

Supplementary Data 7

Supplementary Data 8

Supplementary Data 9

Supplementary Data 10

Supplementary Data 11

Supplementary Data 12

Supplementary Data 13

Supplementary Data 14

Supplementary Data 15

Supplementary Data 16

Supplementary Data 17

Supplementary Data 18

Supplementary Data 19

Supplementary Data 20

Supplementary Data 21

Reporting Summary

### Source data

Source Data

## Data Availability

The data discussed in this publication have been deposited in NCBI’s Gene Expression Omnibus and are accessible through GEO SuperSeries accession number GSE211214. The remaining data are available within the article, Supplementary Information or Source Data file. Source data are provided with this paper.
