# Supplementary material for: Systematic identification of gene combinations to target in innate immune cells to enhance T cell activation
Source: Nat Commun. 2023 Oct 9;14:6295. doi: 10.1038/s41467-023-41792-8 (PMC10562403; doi:10.1038/s41467-023-41792-8)
Supplement: Supplementary file 1 — Supplementary Information [file 41467_2023_41792_MOESM1_ESM.pdf]

Supplementary Fig. 1

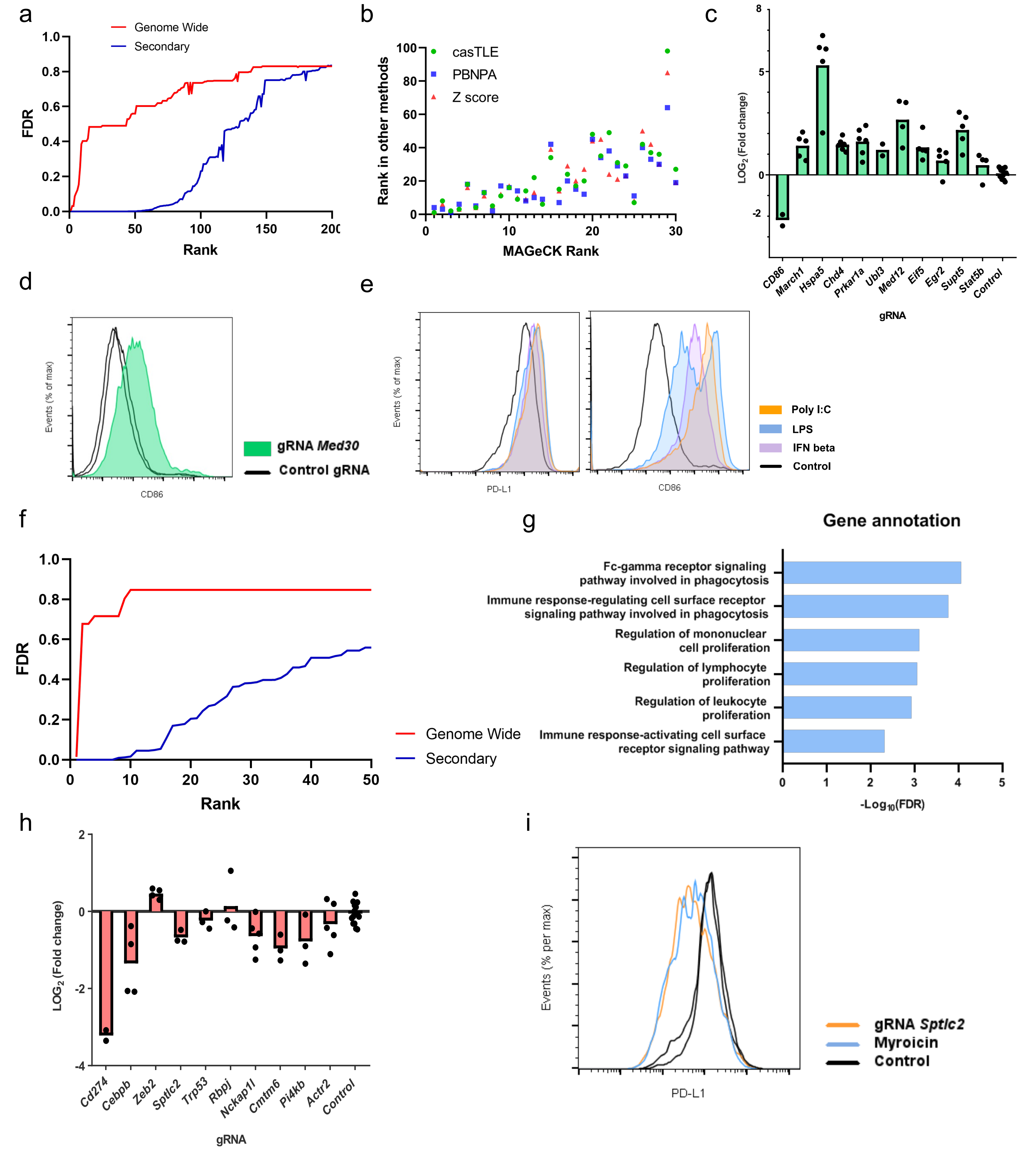

Supplementary Fig. 1: Key regulatory genes that control the expression of CD86 and PD-L1.

a, f, False discovery rates (FDRs) of top-ranked regulators. In (**a)** the CD86 screens' results are presented and in (**f)** the PD-L1 screens' results are presented. The curves of the genome-wide and secondary screens are shown. FDR was calculated using the MAGeCK algorithm. **b,** Top ranked genes of the secondary CD86 screen were computed using several methods. Gene ordered according to MAGeCK (x axis) and ranks obtained by three different methods as indicated (y axis). **c, h**, Quantification of the FACS experiments, n=2-6 biologically independent samples. In (**c)** genes that regulate CD86 and in (**h)** genes that regulate PD-L1. Log2 fold change of the mean fluorescent intensity between targeted gRNA and non-targeting (NT) gRNAs is calculated and each dot represents the value of biological repeat. **d,** FACS analysis of *Med30* targeted cells. **e,** FACS analysis of CD86 and PD-L1 phenotype upon stimulation with TLRs agonists or interferon. CD11c-positive cells were gated and the expression of CD86 and PD-L1 is shown. **g,** Bar plot showing the FDR of GO enrichment analysis of top-ranked positive regulators from the PD-L1 secondary screen. **i,** Analysis of PD-L1 expression in BMDC after targeting *Sptlc2* (orange), or after treatment with myriocin (blue) for 12 hrs, untreated control in black (n = 3 biologically independent samples). Source data are provided as a Source Data file.

Supplementary Fig. 2

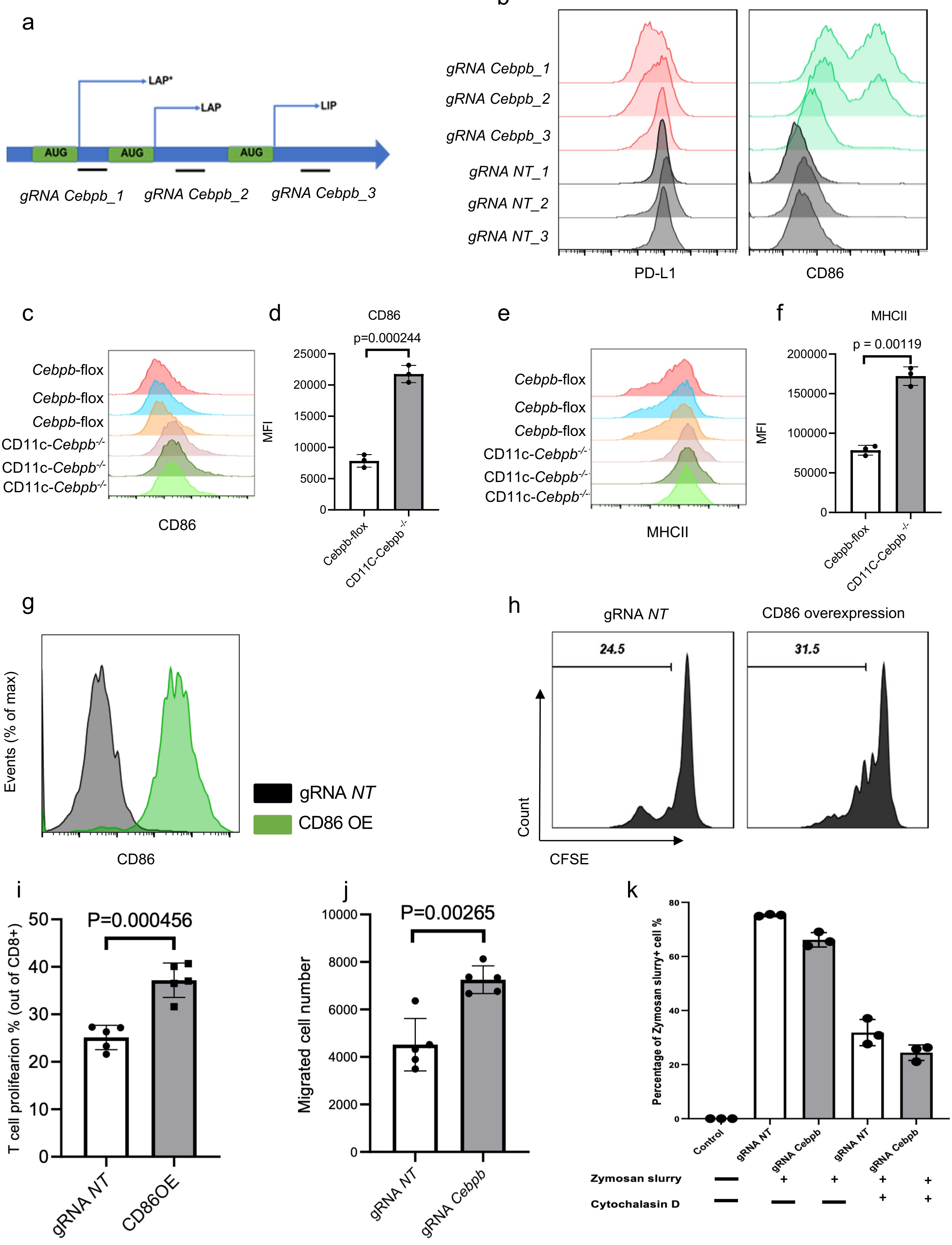

Supplementary Fig. 2: gRNA-*Cebpb* regulates cell migration and expression of co-stimulatory molecules

a, Three translation start sites that form lap*, lap, or lip isoforms. **b,** FACS analysis of cells that express gRNAs as indicated in (**a**). **c***,* FACS analysis showing the expression of CD86 that were recovered from *Cebpb-flox* mice (control) or *CD11c-Cebpb$^{-/-}$* BMDCs*.* **d***,* Quantification of the experiment that is shown in (**c**) (two-tailed unpaired t-test, n = 3 biologically independent samples). **e**, FACS analysis showing the expression of MHC-II in cells that were recovered from *Cebpb-flox* mice (control) or *CD11c-Cebpb$^{-/-}$.* **f***,* Quantification of the experiment that is shown in **(e)** (two-tailed unpaired t-test, n = 3 biologically independent samples). **g,** The expression level of CD86 in BMDCs that were transduced with overexpression plasmid or with control vector. **h**,**i** T cells proliferation after co-culture with BMDCs that overexpress CD86 or a control vector. In **(i)**, quantification of the experiment is presented (two-tailed unpaired t-test. n = 5 biologically independent samples). **j**, Migration assay. Cells were seeded in a transwell and migrated cell numbers are indicated on the y axis (two-tailed unpaired t-test. n = 5 biologically independent samples). **k**, Phagocytosis assay. BMDCs were incubated with florescent Zymosan slurry, with or without cytochalasin D which inhibits phagocytosis. The experiments were done in triplicate. Data are presented as mean values +/- SD in all the graphs. Source data are provided as a Source Data file.

Supplementary Fig. 3

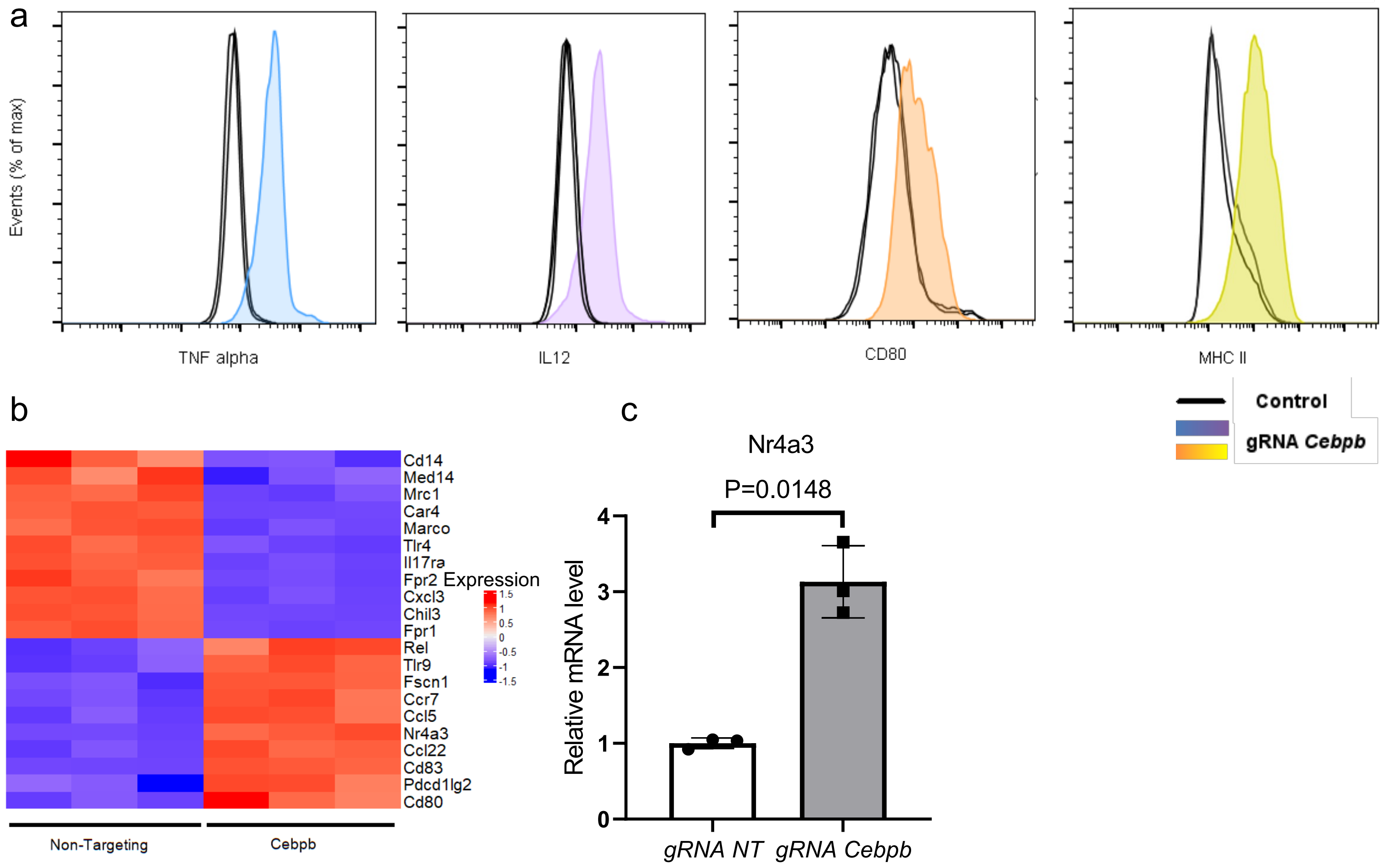

Supplementary Fig. 3: The effect of gRNA-*Cebpb* on gene expression

a, FACS analysis of intracellular TNF (blue), intracellular IL-12 (purple), CD80 (orange), and MHCII (yellow). **b,** Selected differentially expressed genes of bulk RNA-Seq experiment that included cells that express gRNA-*Cebpb* or gRNA-NT (n = 3 biologically independent samples). Rows and columns are hierarchically clustered. Rows are Z-score standardized. Source data are provided as Supplementary Data 5. **c,** QRT-PCR experiment showing the expression level of *Nr4a3* in gRNA-*Cebpb* cells and in gRNA-NT cells. (two-tailed unpaired t-test. n = 3 biologically independent samples). Data are presented as mean values +/- SD. Source data are provided as a Source Data file.

Supplementary Fig. 4

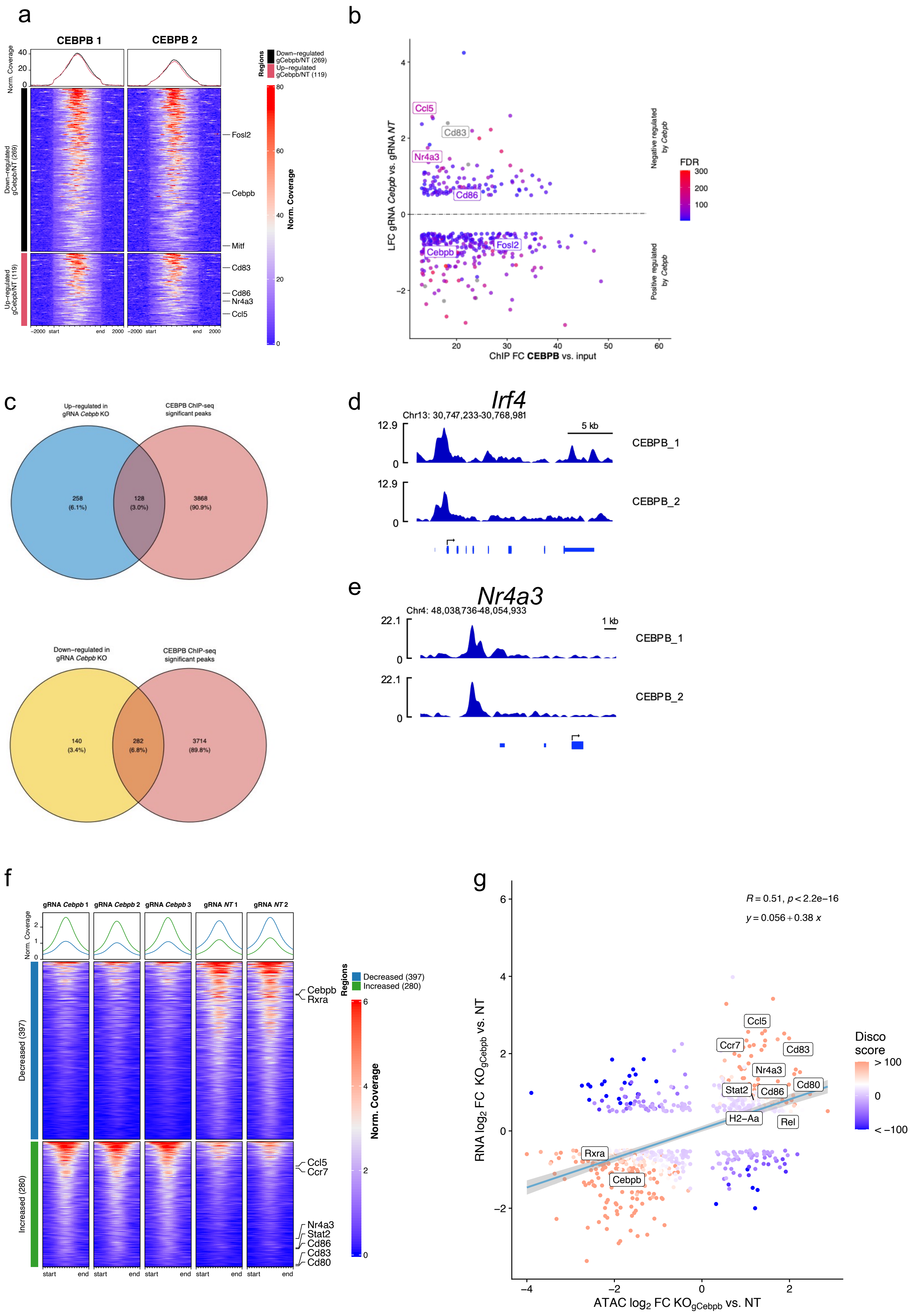

Supplementary Fig. 4: The effect of gRNA-Cebpb on chromatin accessibility and the chromatin binding regions.

a, Heatmap showing ChIP-Seq peaks at sites with enriched (FC > 13, false discovery rate (FDR) < 0.05) CEBPB occupancy and significant (abs(log2FC) > 0.5, FDR < 0.05) differentially expressed genes (DEGs). Source data are provided as Supplementary Data 6. **b**, Dispersion plot showing DEGs (log2FC gRNA-Cebpb versus gRNA-NT, y axis), its significance (FDR color scale), and its signal score (FC) in ChIP-seq data (CEBPB versus input) for genes negative (top) or positive (bottom) regulated by CEBPB. P values were adjusted for FDR using a Benjamini-Hochberg correction. **c**, Venn diagram showing the number of DEGs and genes that were detected in the ChIP-seq experiment. **d**, ChIP-seq pick distribution around the *Irf4* locus. **e**, ChIP-seq pick distribution around the *Nr4a3* locus. **f**, Heatmap showing ATAC-seq peak coverage at genomic loci with significantly increased (green) or decreased (blue) chromatin accessibility in Cebpb targeted cells versus. non-targeted cells (NT). Genomic regions were selected based on their concordance with RNA-seq data. Technical repeats are shown. P values were calculated using two-tailed Wald test, and Benjamini-Hochberg was used to calculate the FDRs. **g**, Dispersion plot showing the concordance between ATAC-seq and RNA-seq data. Genes with high concordance are shown in red and genes with discordance are in blue. Pearson correlation test is shown. Source data are provided as Supplementary Data 7.

Supplementary Fig. 5

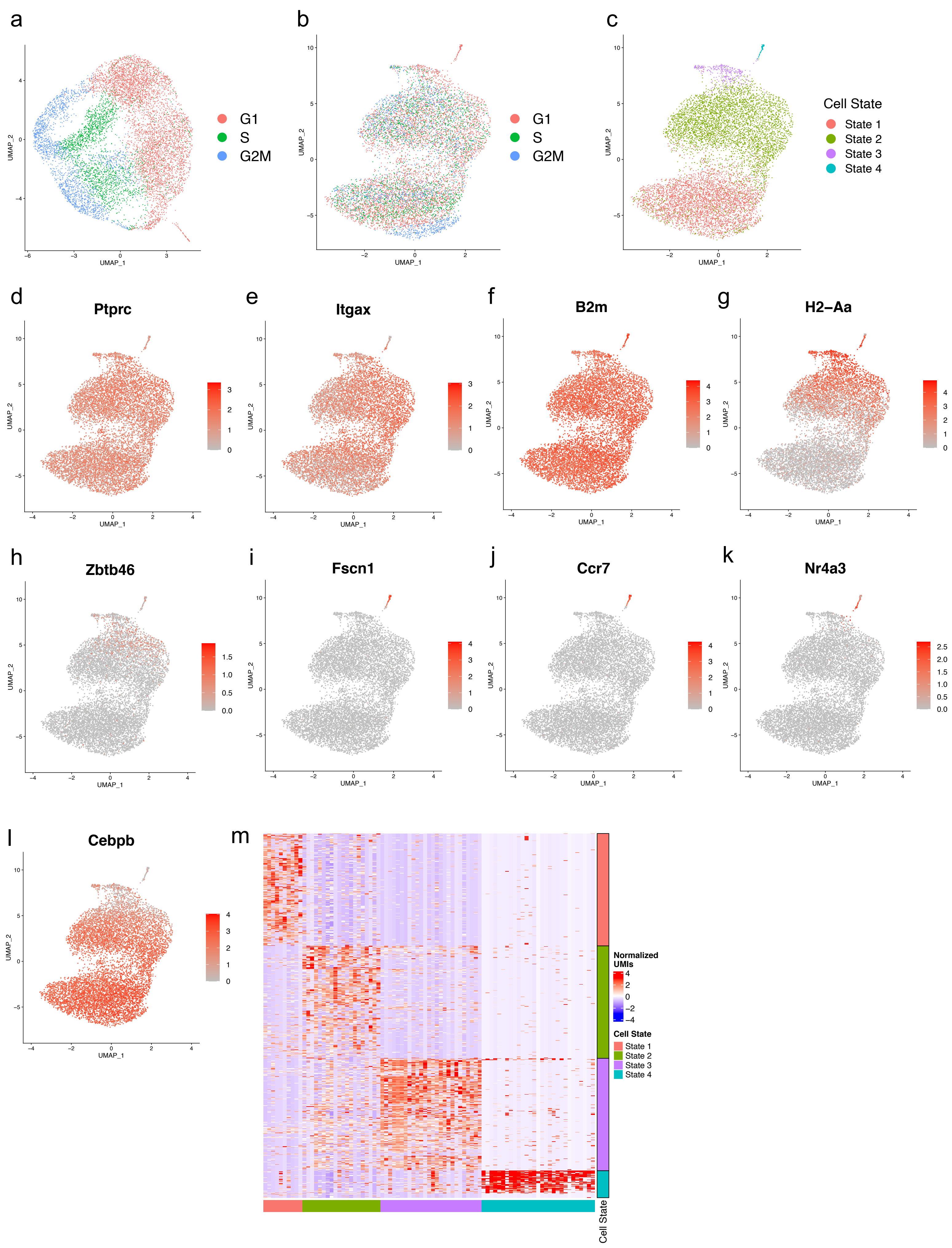

Supplementary Fig. 5: Four cell states can be detected in BMDCs.

Single-cell RNA-seq experiment, 9219 cells are shown. Cells were infected with lentiviruses that encode gRNA-*Cd86*, gRNA-*Cd274* or gRNA-NT. **a**, Uniform manifold approximation and projection (UMAP) showing cell cycle distribution **b**, Cells shown in (**a**) after cell cycle regression. **c**, Each cell in the UMAP is colored according to the assignment of cell state. **d-l**, UMAP is colored based on the expression level of a selected set of genes. The expression level is indicated on the right and the gene name on the top of each chart. **m,** Expression of genes that were selected for the signature of the cell states across the dataset. Rows represent cells and columns represent genes that were included in the signatures. The assignment of cells to cell states is shown in the bar on the right, and the assignment of genes to cell state signature is shown in the bar on the bottom. A random subset of 250 cells was selected for cell states 1, 2 and 3. UMIs are log normalized.

Supplementary Fig. 6

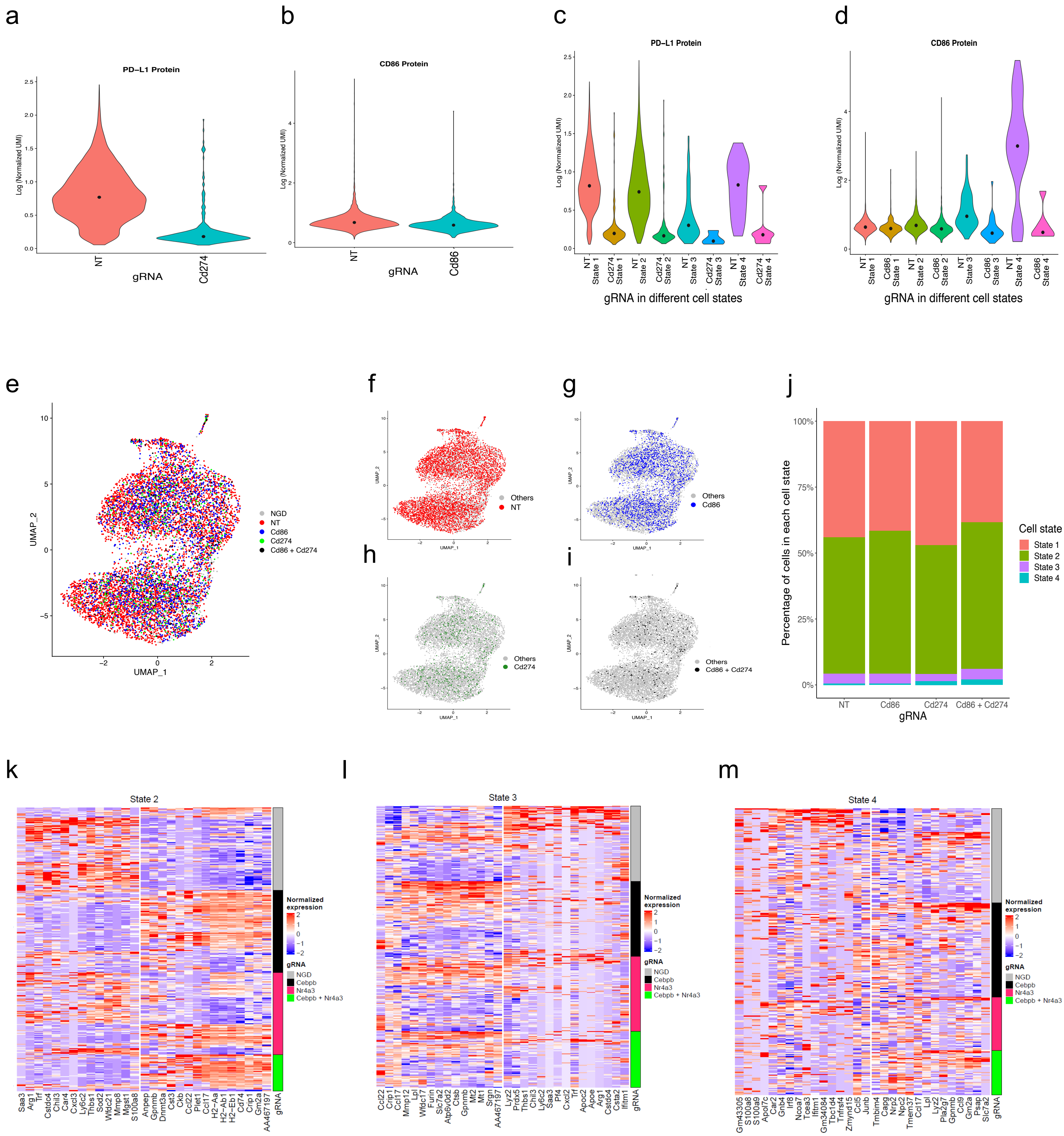

Supplementary Fig. 6: The effect of perturbations on gene expression and protein expression.
a, b, Violin plots showing the relative expression of PD-L1 (**a**) and CD86 (**b**) across cells that express gRNA as indicated in the bottom. NT - non-targeting **c, d**, expression of PD-L1 (**c**) and CD86 (**d**) in each cell state. **e-i**, Uniform manifold approximation and projection (UMAP) of the single cell RNA-seq experiment. Cells are colored according to the identity of the gRNA that was detected. NGD - no guide detected. **j**, Distribution of cells with different gRNAs across the four cell states. **k-m**, Heatmaps showing top differentially expressed genes between different gRNAs in each cell state. Log normalized UMIs were Z-score standardized across columns (**k**) state 2, (**l)** state 3, (**m**) state 4.

Supplementary Fig. 7

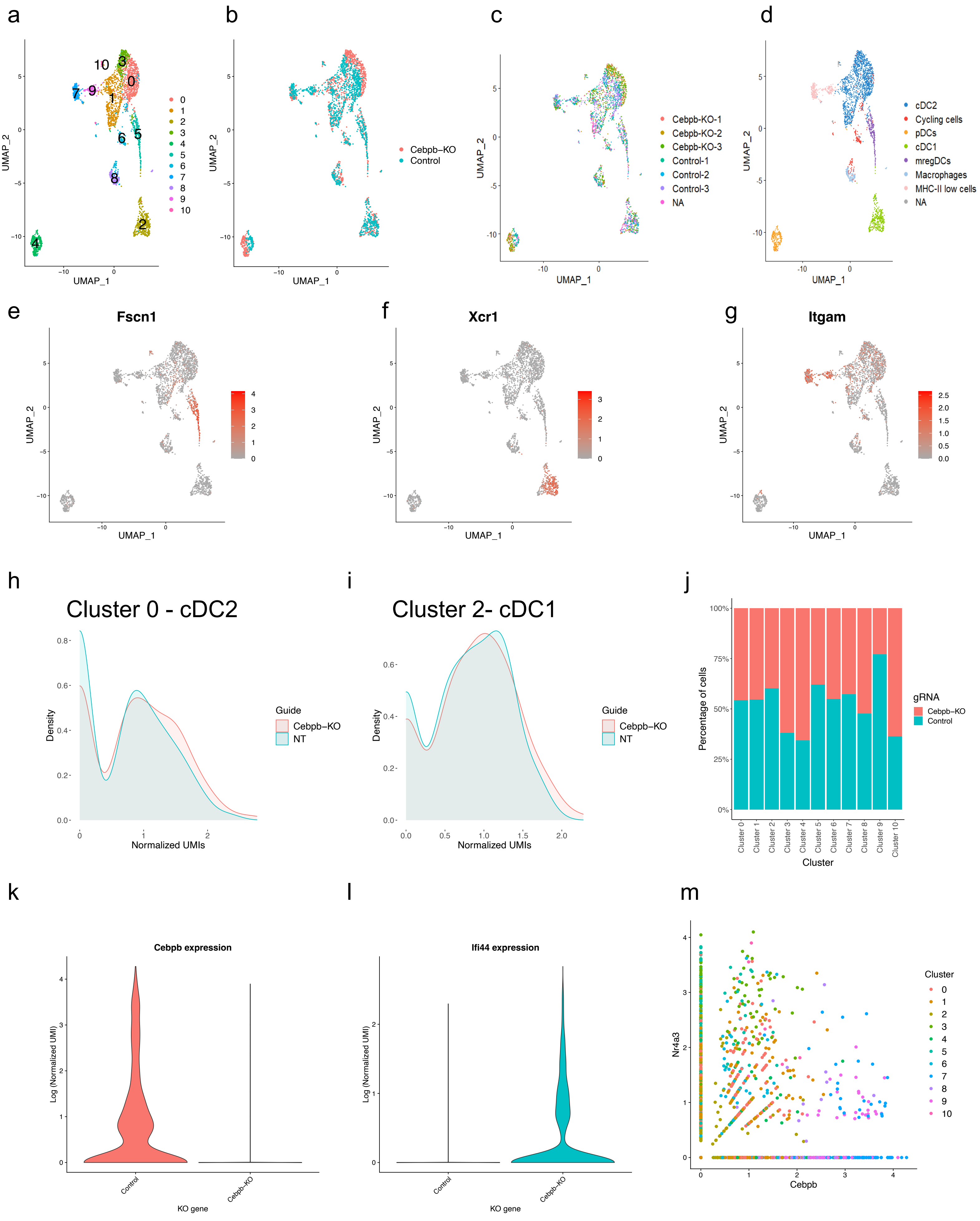

Supplementary Fig. 7: Expression profile of CD11c positive cells from Cebpb- knock out mice

Single-cell RNA-seq of CD11c positive cells from spleens of Cre-CD11c Cebpb-fl/fl and control mice. 2901 cells are shown. **a**, Uniform manifold approximation and projection (UMAP) showing different cell clusters. **b-c**, UMAP showing the distribution of cells across different conditions (**b**) and mice (**c**). **d**, UMAP is colored based on cell annotation. **e-g**, UMAP is colored based on gene expression (normalized UMIs). The expression level is indicated on the right and the gene name is at the top of each chart. **h-i**, The distribution of *Cd86* expression in Cre-CD11c Cebpb-fl/fl (red) or Cebpb-fl/fl (blue) cluster 0 cells (**h**) or cluster 2 cells (**i**). **j**, The relative distribution of each condition in each cluster. **k-l**, violin plot showing *Cebpb* (**k**) or *ifi44* (**l**) gene expression. **m**, The expression of *Cebpb* (x axis) and *Nr4a3* (y axis) in the control sample. Cells are colored according to the cluster. Source data are provided as Supplementary Data 9.

Supplementary Fig. 8

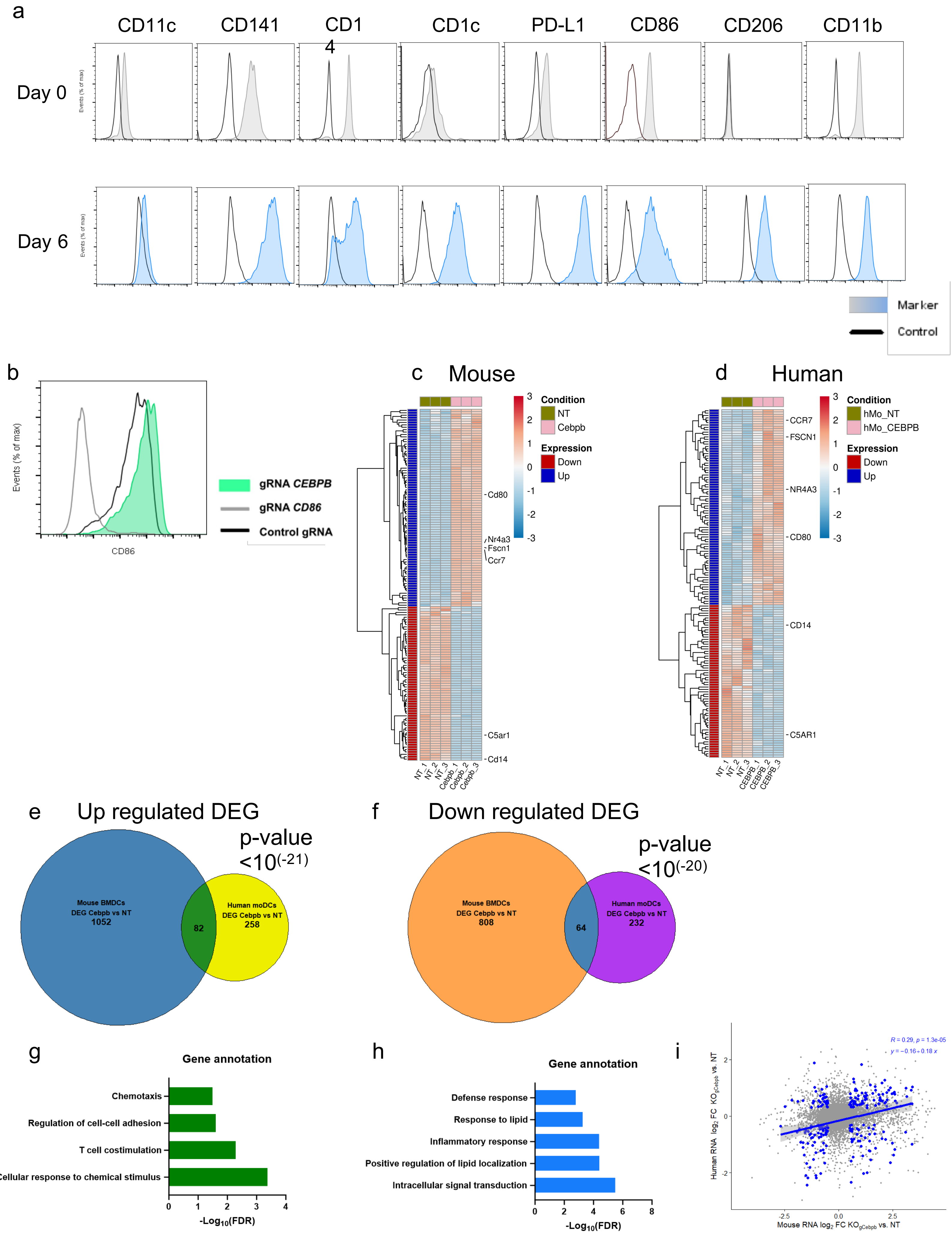

Supplementary Fig. 8: Targeting human monocytes.

CD14-positive human monocytes were isolated from peripheral blood and nucleofected with CAS9 protein and gRNAs*.* **a**, FACS analysis of markers of myeloid cell types. In the upper panels, FACS analysis was performed immediately after cell isolation. In the lower panels, FACS analysis of GMCSF and IL4-treated cells was performed six days after isolation. Unstained control is colored in black. **b,** FACS analysis showing the expression of CD86. gRNA-NT is colored in black and gRNA-CD86 is colored in gray. We performed three repeats of the experiment. **c,d**, Bulk RNA-seq experiment of mouse BMDCs (**c**) or human monocytes (**d**). The expression level of differentially expressed genes (DEGs) between gRNA-CEBPB and gRNA-NT that overlap between the human and mouse data are shown in the heatmap. **e**. A Venn diagram showing the number of DEGs that are upregulated in both humans and mice data. The p-value was calculated based on a hypergeometric test. **f**, Venn diagram showing the number of DEGs that are downregulated in humans and mice. P-value was calculated based on a hypergeometric test. **g**, GO annotation analysis of shared upregulated genes. **h**, GO annotation analysis of shared downregulated genes. **i** All genes with a unique ortholog are shown in gray. The shared DEGs in both experiments are shown in blue. Linear regression analysis for shared DEGs is shown. X axis - Log2 fold change (cells with gRNA-CEBPB / cells with gRNA-NT) for mouse bulk RNA-seq experiment. Y axis - Log2 fold change (cells with gRNA-*cebpb* / cells with gRNA-NT) for human bulk RNA-seq experiment. Source data are provided as Supplementary Data 5 and 10.

Supplementary Fig. 9

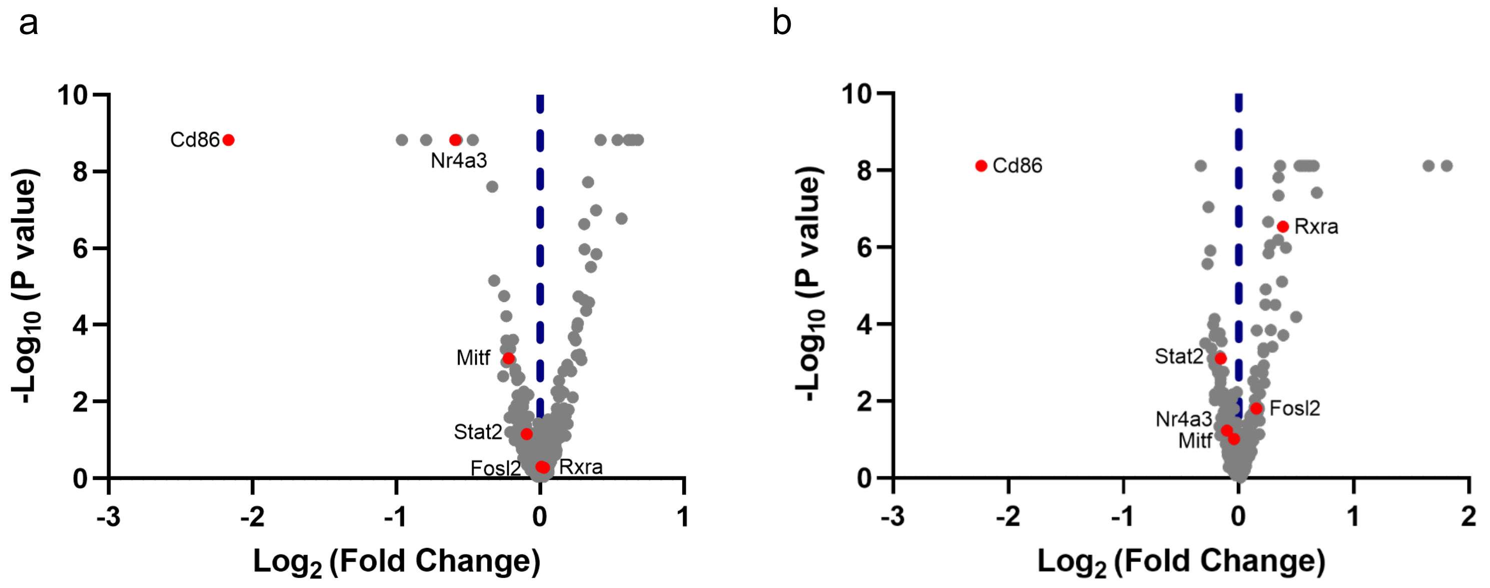

Supplementary Fig. 9: CRISPR screen in CAS9 Cd11c-Cre Cebpb(fl/fl) and control CD86 regulators.
a-b, CRISPR screen in bone marrow differentiated cells from CAS9 Cd11c-Cre Cebpb(fl/fl) mice in **a** or control mice in **b**. The screens included 10 gRNA for each differentially expressed gene in the Cebpb versus non-targeting bulk RNA-seq experiments. Volcano plots show the results for each gene in the screen. The log-transformed p-values are calculated based on the MAGeCK algorithm (y axis). Source data are provided as Supplementary Data 12.

Supplementary Fig. 10

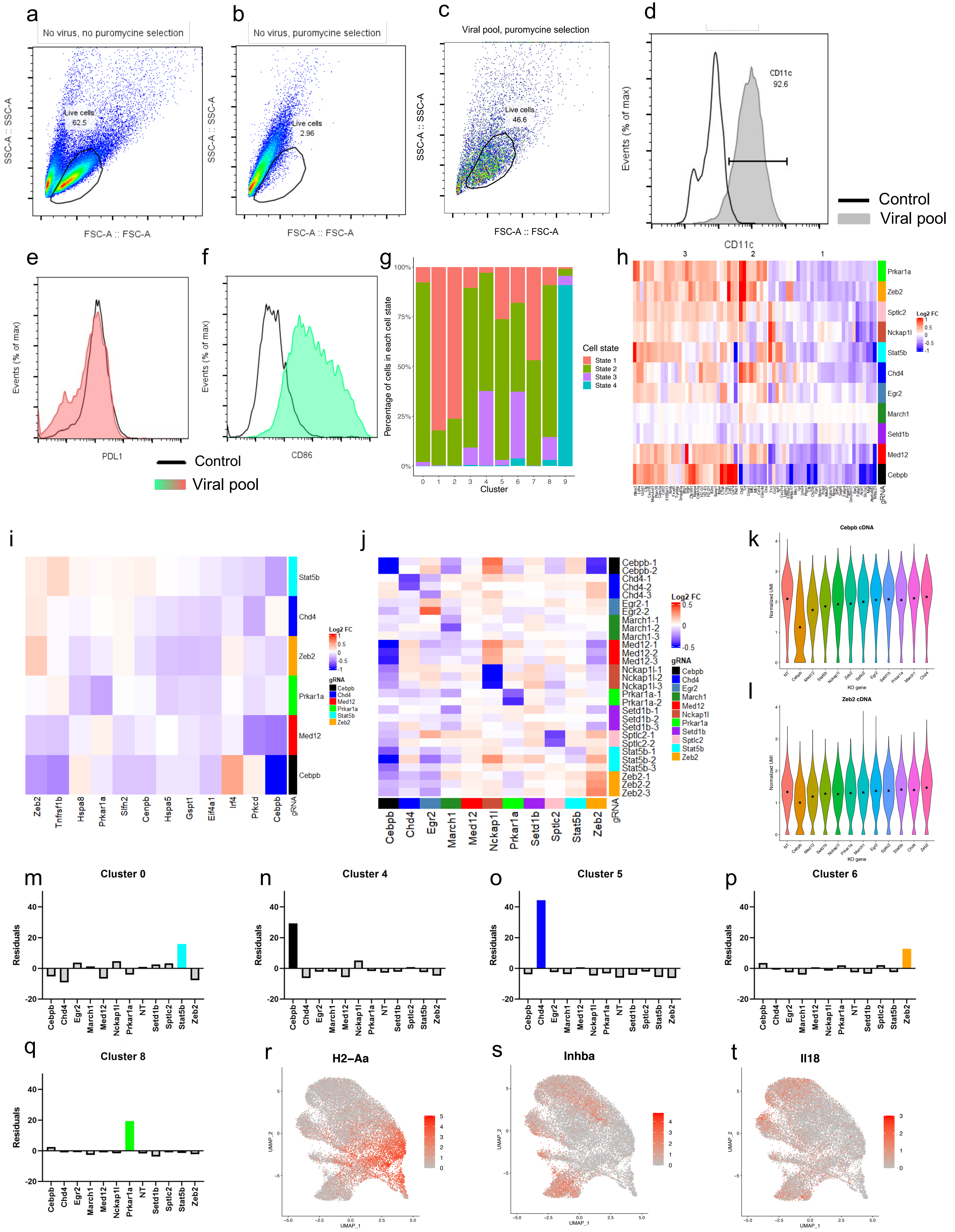

Supplementary Fig. 10: Single-cell RNA-seq experiment setting and top expressed genes in perturb cells.

a-f, FACS analysis of the Perturb-seq experiment that is shown in Fig.4. Infected cells were selected with puromycin **(a-c)**. The expression of CD86 (**f**) and PD-L1 (**e**) in CD11c positive cells (**d**). **g,** Distribution of cell states in each cluster. **h,** Differentially expressed genes (compared to cells with NT-gRNA) that were shared across several perturbations. Genes with FDR<0.05 that were shared across four or more perturbations are shown. Rows are hierarchically clustered, and columns are K-means (k=3) clustered. **i**, Differentially expressed genes that were shared across selected perturbations, and were highly ranked as regulators (FDR<0.05) in the secondary CRISPR screens for CD86 or PD-L1. Fold-change relative to cells with NT-gRNA is shown. **j**, The effect of each gRNA on the expression of all the perturbed genes. Rows represent gRNAs, columns show gene expression of the respective genes. Fold-changes relative to cells that express NT-gRNA are shown. **k**, The effect of different perturbations on the expression of *Cebpb*, y axis - log-normalized UMI. **l**, The effect of different perturbations on the expression of *Zeb2*, *y* axis - log-normalized UMI. **m-q**, Gene perturbations enrichment in clusters that are indicated at the top. The standardized residual values of a chi-squared test are shown on the y axis and perturb genes are shown on the x axis. **r-t**, Uniform manifold approximation and projection (UMAP) showing the normalized expression of selected genes. Source data are provided as Supplementary Data 2,4,16,17.

Supplementary Fig. 11

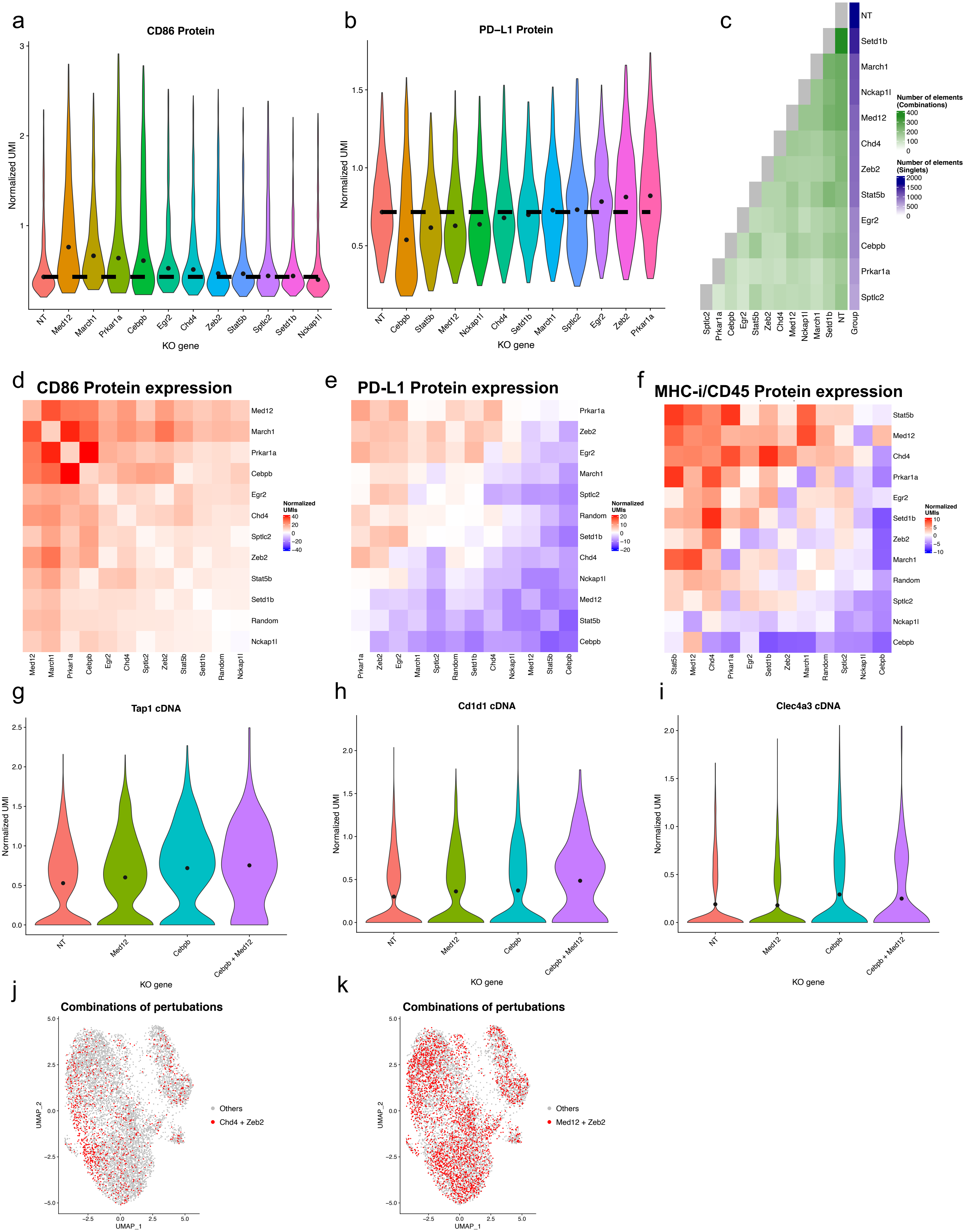

Supplementary Fig. 11: The effects of combinations of perturbations on immune cells.

a,d The expression of CD86 is based on the barcoded antibody that was detected in the single-cell RNA-seq (scRNA-seq) experiment. In (**a**)**,** violin plots for every perturbed gene are presented. The dots represent the median value and the dashed line shows the median normalized UMI's of CD86 in cells that express gRNA-NT. The top and bottom two percent of outlier cells were removed. UMIs are centered log-ratio normalized. In (**d**) the value of CD86 for each combination of targeted genes is shown. Relative counts were calculated, and the values were normalized to cells that express gRNA-NT. **b,e** same as (**a**) and (**d**) using the detected signal from the PD-L1 antibody. **c**, The number of cells for every single perturbation is indicated on the right bar and the number of cells that carry each combination of perturbations is shown on the left diagram. Source data are provided as Supplementary Data 15. **f,** The expression of a mix of barcoded antibodies that bind CD45 and MHC-I that were detected in the scRNA-seq experiment. Relative counts were calculated, and the values were normalized to cells that express gRNA-NT. **g-i,** The expression of genes in cells that express gRNA-NT, gRNA-*Cebpb*, gRNA-*Med12* or gRNA-*Cebpb* gRNA-*Med12*. (**g**) *Tap1*, (**h)** *Cd1d1*, (**i**)**,** *Clec4a3*. **j,** T cells that were incubated with BMDCs that express gRNA-*Chd4* gRNA-*Zeb2* are colored in the UMAP. **k,** T cells that were incubated with BMDCs that express gRNA-*Zeb2* gRNA-*Med12* are colored in the UMAP.

Supplementary Fig.12

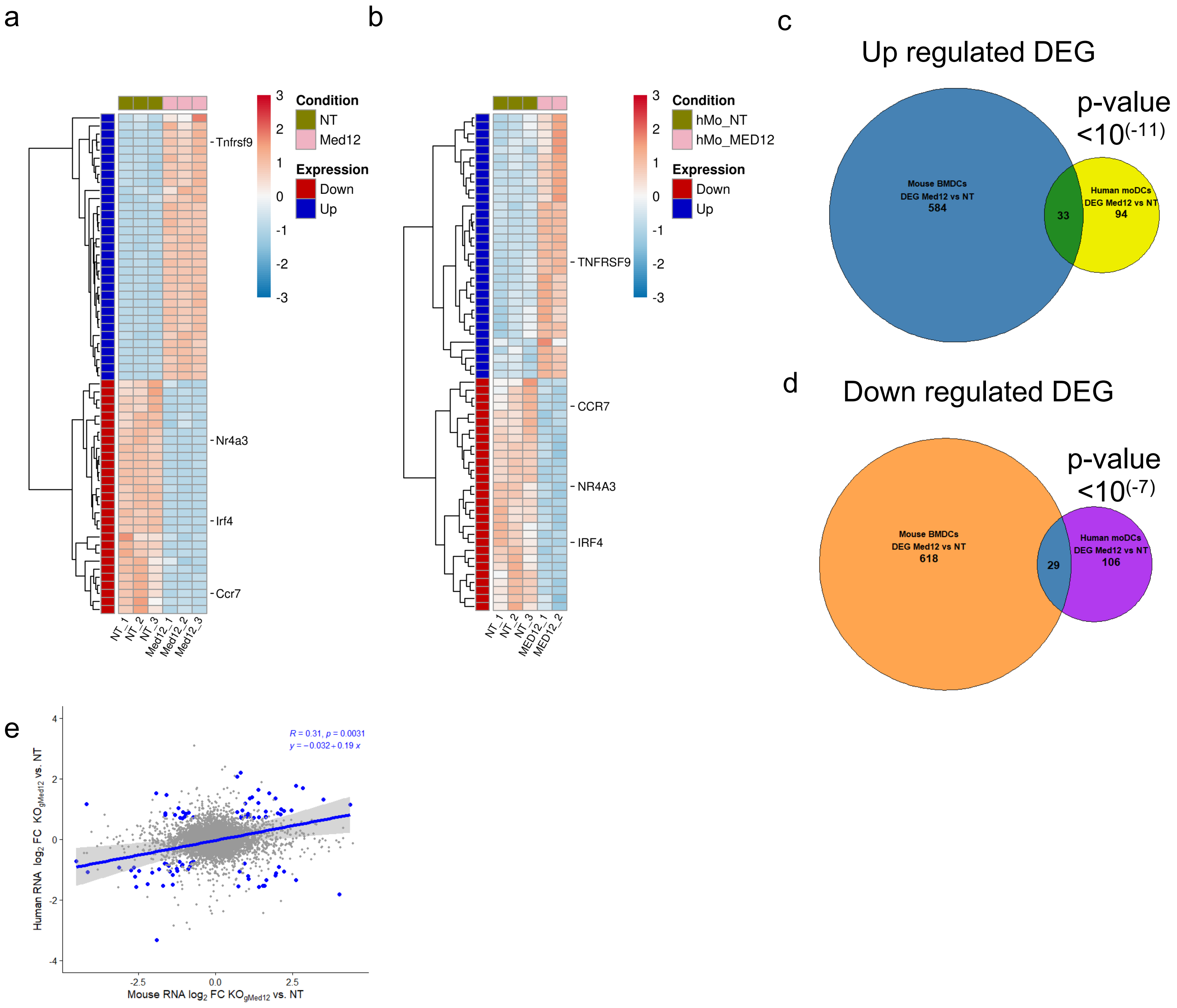

Supplementary Fig. 12: a,b, Bulk RNA-seq experiment in mouse BMDCs (**a**) or human monocytes (**b**). The expression level of Differentially expressed genes (DEGs) between gRNA-Med12 and gRNA-NT that overlap between the human and mouse data are shown in the heatmap. **c**. Venn diagram showing the number of DEGs that are upregulated in both human and mouse data. The p-value was calculated based on a hypergeometric test. **d**, Venn diagram showing the number of DEGs that are downregulated in both human and mouse data. P-value was calculated based on a hypergeometric test. **e** All genes with a unique ortholog are shown in gray. The shared DEGs in both experiments are shown in blue. Linear regression analysis for shared DEG is shown. X axis - Log2 fold change (cells with gRNA-CEBPB / cells with gRNA-NT) for mouse bulk RNA-seq experiment. Y axis - Log2 fold change (cells with gRNA-*cebpb* / cells with gRNA-NT) for human bulk RNA-seq experiment. Source data are provided as Supplementary Data 18 and 19.

Supplementary Fig. 13

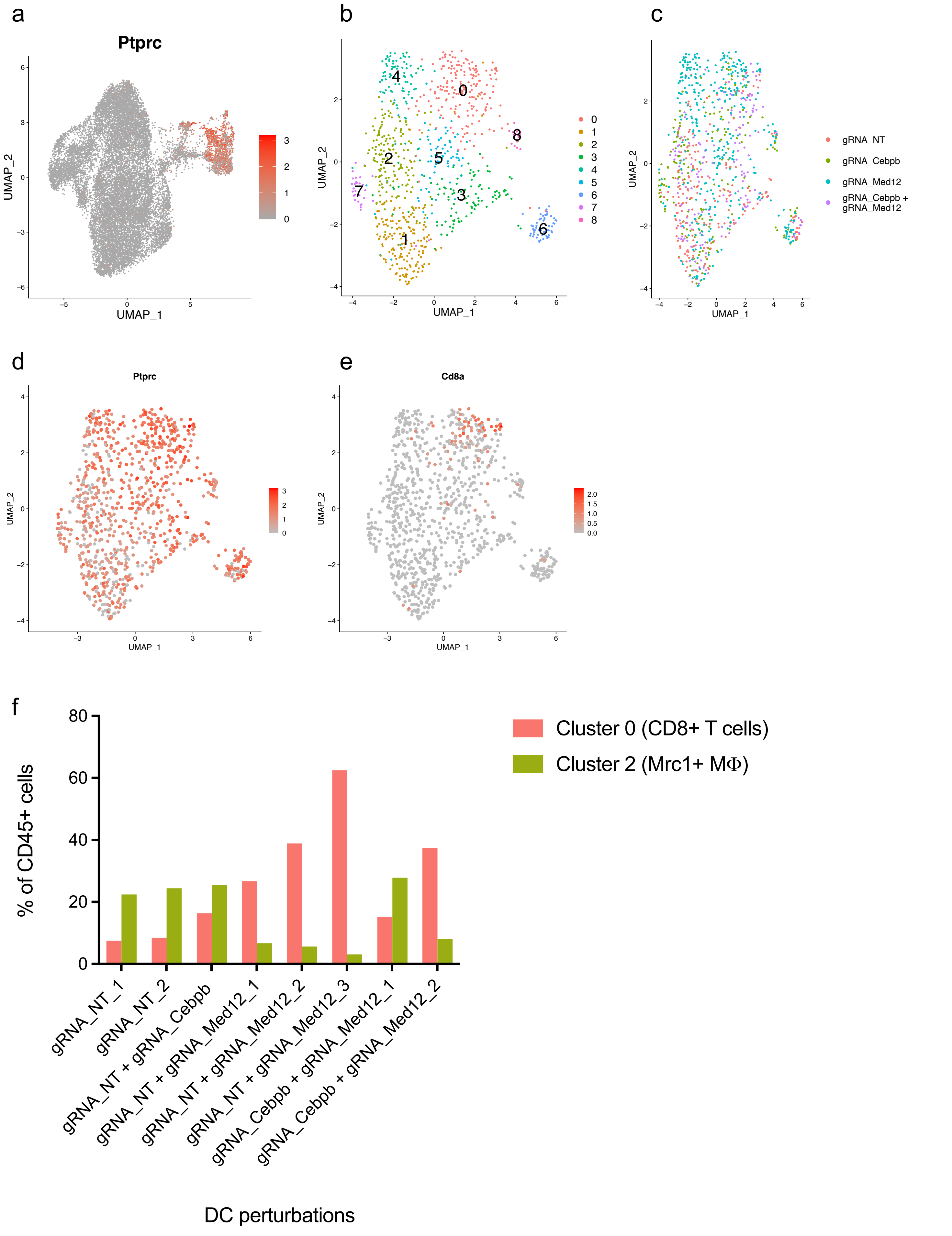

Supplementary Fig. 13: The effect of adoptive transfer of perturbed BMDCs on tumor-associated immune cells.

Adoptive cell transfer of *gRNA-NT*, *gRNA-Cebpb*, *gRNA-Med12*, or *gRNA-Cebpb gRNA-Med12* BMDCs to B16 tumor-bearing mice. **a**, Uniform manifold approximation and projection (UMAP) showing CD45 (*Ptprc*) expression level. **b**, UMAP showing the analysis of the CD45 positive subpopulation. 920 cells are shown. **c**, Each cell in the UMAP is colored according to the BMDCs perturbation. **d-e**, UMAP is colored according to the expression (normalized UMIs) of *Cd45* or *Cd8a*. The expression level is indicated on the right. **f**, The percentage of CD8 T cells (cluster 0) and CD206 positive macrophages (cluster 2) from the CD45 positive cells in each mouse in the experiment. Source data are provided as Supplementary Data 20.

Supplementary Table 1: Nepa 21 settings for monocytes nucleofection

Poring pulse						Transfer pulse					
V	Length (ms)	Interval (ms)	.No	D.Rate (%)	Polarity	V	Length (ms)	Interval (ms)	.No	D.Rate (%)	Polarity
275	1	50	2	40	+	20	50	50	5	40	-/+
