# Supplementary material for: Systematic identification of gene combinations to target in innate immune cells to enhance T cell activation
Source: Nat Commun. 2023 Oct 9;14:6295. doi: 10.1038/s41467-023-41792-8 (PMC10562403; doi:10.1038/s41467-023-41792-8)
Supplement: Supplementary file 3 — Description of Additional Supplementary Files [file 41467_2023_41792_MOESM3_ESM.pdf]

Description of Additional Supplementary Files

Supplementary Data 1 - CD86 genome-wide screen: Three sheets that include raw reads and results of MAGeCK analysis for genes and for individual gRNA.

Supplementary Data 2 - CD86 secondary screen: Four sheets that include raw reads, results of MAGeCK analysis for genes and for individual gRNA and gRNA sequences that were included in the secondary library.

Supplementary Data 3 - PDL1 genome-wide screen: Three sheets that include raw reads and results of MAGeCK analysis for genes and for individual gRNA.

Supplementary Data 4 - PDL1 Secondary Screen: Four sheets that include raw reads, results of MAGeCK analysis for genes and for individual gRNA and gRNA sequences that were included in the secondary library.

Supplementary Data 5 - Bulk RNA-seq gRNA-*Cebpb* vs gRNA-NonTarget: Results of bulk RNA-seq analysis from three repeats of *Cebpb* targeted BMDCs and three repeats of samples from BMDCs that were infected with non-targeting gRNA. Results were generated with DESeq2.

Supplementary Data 6 - CEBPB ChIP-seq: Results of ChIP-seq analysis showing the location of the peaks that relate to CEBPB binding. Peak signal values represent the signal from anti-CEBPB precipitation over the signal from the input.

Supplementary Data 7 - ATAC-seq of *Cebpb* targeted cells: Results of ATAC-seq analysis showing the chromosomal accessible regions in *Cebpb* targeted cells compared to cells that express non-targeting gRNA.

Supplementary Data 8 - Bulk RNA-seq of *Cebpb* and *Nr4a3* targeted BMDCs: The first sheet shows bulk RNA-seq of cells that were infected with gRNA-NT, *Cebpb*-gRNA, *Nr4A3*-gRNA and the double targeted *Cebpb*- gRNA *Nr4A3*-gRNA. The second sheet shows the cluster of differentially expressed genes between gRNA-NT and *Cebpb*-gRNA, related to Fig. 3c. Results were generated with DESeq2.

Supplementary Data 9 - Single-cell RNA-seq from splenocytes that were recovered from Cebpb KO mice: Differentially expressed genes between CD11c positive splenocytes from Cd11c-CRE Cebpb (fl/fl) mice and control mice, in each cluster. Results were generated by FindMarkers function in Seurat.

Supplementary Data 10 - Bulk RNA-seq of human monocyte: Results of bulk RNA-seq from three samples of *Cebpb* targeted human differentiated monocytes and three samples of human differentiated monocytes that express non-targeting gRNA. Results were generated with DESeq2.

Supplementary Data 11 - Genome-wide CRISPR screen using BMDCs from Cebpb KO mice: Genome-wide CRISPR screen, to find CD86 regulators. The screen was done using BMDCs from Cd11c-CRE Cebpb (fl/fl). Three sheets that include raw reads and results of MAGeCK analysis for genes and for individual gRNA.

Supplementary Data 12 - Small-scale CRISPR screen in Cebpb KO or control BMDCs: Results of two CRISPR screens to find CD86 regulators – gRNA targeting differentially expressed genes that are shown in Supplementary Data 5 were included. In the first screen BMDCs from Cd11c-CRE Cebpb (fl/fl) mice were used and in the second screen BMDCs from control mice were used. For each screen, raw reads and MAGeCK analysis results for genes and for individual gRNA are provided. The library of gRNA is provided in a separate spreadsheet.

Supplementary Data 13 - Motif analysis of accessible regions in *Cebpb* targeted cells: The analysis was done using the chromVAR tool.

Supplementary Data 14 - Motif analysis of CEBPB binding sites: The analysis was done using Homer, and significantly enriched motifs were calculated using the Fisher Exact test.

Supplementary Data 15 - Numbers of cells with each perturbation: Numbers of high-quality cells for each perturbation or combination of perturbations are shown. Relate to Fig. 4.

Supplementary Data 16 - High MOI perturb-seq experiment differential expressed genes in each perturbation: Differentially expressed genes between perturbations. Results were generated by FindMarkers function in Seurat. Raw data for Supplementary Fig. 10h in the second sheet.

Supplementary Data 17 - High MOI perturb-seq experiment differential expressed genes in each cluster: Differentially expressed genes between clusters. Results were generated by FindMarkers function in Seurat.

Supplementary Data 18 - Bulk RNA-seq of *Med12* targeted mouse BMDCs: Analysis output of RNA-seq of *Med12* targeted BMDCs and cells that were infected with non-targeting gRNA. Results were generated by DESeq2.

Supplementary Data 19 - Bulk RNA-seq *Med12* targeted human monocytes: Analysis output of RNA-seq of *Med12* targeted human differentiated monocytes compared to cells that express non-targeting gRNA. Results were generated by DESeq2.

Supplementary Data 20 – Single-cell RNA-seq of B16 tumors: ScRNA-seq of digested B16 tumors after adoptive transfer of targeted BMDCs. Results were generated by FindMarkers function in Seurat.

Supplementary Data 21 - gRNA sequences: Additional gRNAs that were used in this study and are not shown in Supplementary Data 2 and Supplementary Data 4.
