# Supplementary material for: Systematic identification of gene combinations to target in innate immune cells to enhance T cell activation
Source: Nat Commun. 2023 Oct 9;14:6295. doi: 10.1038/s41467-023-41792-8 (PMC10562403; doi:10.1038/s41467-023-41792-8)
Supplement: Supplementary file 25 — Reporting Summary [file 41467_2023_41792_MOESM25_ESM.pdf]

nature portfolio

Corresponding author(s): Oren Parnas, Yehuda Shovman

Last updated by author(s): 2023/07/22

Reporting Summary

Nature Portfolio wishes to improve the reproducibility of the work that we publish. This form provides structure for consistency and transparency in reporting. For further information on Nature Portfolio policies, see our Editorial Policies and the Editorial Policy Checklist.

Statistics

For all statistical analyses, confirm that the following items are present in the figure legend, table legend, main text, or Methods section.

n/a	Confirmed	
☐	☒	The exact sample size (*n*) for each experimental group/condition, given as a discrete number and unit of measurement
☐	☒	A statement on whether measurements were taken from distinct samples or whether the same sample was measured repeatedly
☐	☒	The statistical test(s) used AND whether they are one- or two-sided<br>*Only common tests should be described solely by name; describe more complex techniques in the Methods section.*
☒	☐	A description of all covariates tested
☐	☒	A description of any assumptions or corrections, such as tests of normality and adjustment for multiple comparisons
☐	☒	A full description of the statistical parameters including central tendency (e.g. means) or other basic estimates (e.g. regression coefficient) AND variation (e.g. standard deviation) or associated estimates of uncertainty (e.g. confidence intervals)
☐	☒	For null hypothesis testing, the test statistic (e.g. *F*, *t*, *r*) with confidence intervals, effect sizes, degrees of freedom and *P* value noted<br>*Give P values as exact values whenever suitable.*
☒	☐	For Bayesian analysis, information on the choice of priors and Markov chain Monte Carlo settings
☒	☐	For hierarchical and complex designs, identification of the appropriate level for tests and full reporting of outcomes
☒	☐	Estimates of effect sizes (e.g. Cohen's *d*, Pearson's *r*), indicating how they were calculated

Our web collection on statistics for biologists contains articles on many of the points above.

Software and code

Policy information about availability of computer code

Data collection	We did not collect any data, this is not applicable for our study.
Data analysis	General: Bcl2fastq (v 2.20.0.422), Cutadapt (v 2.10, v 3.4), R (v 4.0.2), python (v 2.7.18, v 3.9.1)<br>CRISPR-CAS9 screens: bowtie1 (v 1.3.1), Mageck (v 0.5.9.4), casTLE (v 1.0), PBNPA (v 0.0.3)<br>Bulk RNA-seq: TopHat (v 2.1.1), htseq-count (v 0.6.0), STAR (v 2.7.3), salmon (v 0.14.0), DESeq2 (v 1.26.0)<br>Single-cell RNA-seq: Cellranger (v 4.0.0), CITE-seq-count (v 1.4.3), Seurat (v 4.0.4), UCell (v 1.1)<br>ATAC-seq and ChIP-seq: trim-galore (v 0.6.7), hisat2 (v 2.2.1), picard (v 2.26.10), deeptools (v 3.5.1), profileplyr (v 1.14.1), macs (v 2.2.7.1), ChIPseeker (v 1.34.1), chromVAR (v 1.20.2), disco (v 0.6)

For manuscripts utilizing custom algorithms or software that are central to the research but not yet described in published literature, software must be made available to editors and reviewers. We strongly encourage code deposition in a community repository (e.g. GitHub). See the Nature Portfolio guidelines for submitting code & software for further information.

Data

Policy information about availability of data

All manuscripts must include a data availability statement. This statement should provide the following information, where applicable:

- Accession codes, unique identifiers, or web links for publicly available datasets
- A description of any restrictions on data availability
- For clinical datasets or third party data, please ensure that the statement adheres to our policy

Data was deposited in GEO under the accession number GSE211214

Human research participants

Policy information about studies involving human research participants and Sex and Gender in Research.

Reporting on sex and gender	We have used several blood samples (PBMCs) from healthy donors that confirmed that the samples will be used for research.
Population characteristics	Healthy donors
Recruitment	The recruitment is random
Ethics oversight	Human peripheral blood mononuclear cells (PBMCs) were obtained from the Hebrew University-Hadassah Medical Center (Jerusalem, Israel). The studies were conducted in accordance with ethical guidelines (0268-19-HMO Declaration of Helsinki). The study was approved by the Hadassah Medical Center committee for human experiments (Helsinki Committee).

Note that full information on the approval of the study protocol must also be provided in the manuscript.

Field-specific reporting

Please select the one below that is the best fit for your research. If you are not sure, read the appropriate sections before making your selection.

- [x] Life sciences
- [ ] Behavioural & social sciences
- [ ] Ecological, evolutionary & environmental sciences

For a reference copy of the document with all sections, see nature.com/documents/nr-reporting-summary-flat.pdf

Life sciences study design

All studies must disclose on these points even when the disclosure is negative.

Sample size	We performed at least 3 biological repeats and at least 3 technical repeat in each phenotypic experiment, as indicated in the legend. The number of repeats were determine according to the consistency in the results.
Data exclusions	We did not exclude data, except single-cell RNA-seq experiments that had technically failed
Replication	We repeated every experiment several times, unless otherwise is indicated in the legend, and documented all the replications as described above in "sample size" section. All the experiments are reproducible.experiments are reproducible.
Randomization	There is no rationale in performing randomization in this kind of experiments
Blinding	Different students performed the experimental part and the computational part of the study. However, there is no rationale in blinding these experiments

Reporting for specific materials, systems and methods

We require information from authors about some types of materials, experimental systems and methods used in many studies. Here, indicate whether each material, system or method listed is relevant to your study. If you are not sure if a list item applies to your research, read the appropriate section before selecting a response.

Materials & experimental systems

n/a	Involved in the study	
	x	Antibodies
	x	Eukaryotic cell lines
x		Palaeontology and archaeology
	x	Animals and other organisms
x		Clinical data
x		Dual use research of concern

Methods

n/a	Involved in the study	
	x	ChIP-seq
	x	Flow cytometry
x		MRI-based neuroimaging

Antibodies

Antibodies used	Screens: CD11c (BLG-117310), CD274 (BLG-124307) and CD86 (BLG-159203).<br>Single gRNA validations: CD11c (BLG-117322), CD274 (BLG-124307), CD86 (BLG-105012).<br>Additional markers: CD80 (BLG-104708), MHCI (H-2Kb) (BLG-116505), CCR7 (CD197) (BLG-120107), MHCII I-A/I-E (BLG-107606), IL-12/IL-23 p40 (BLG-505204) and TNF-α (BLG-506306).<br>Hashtag-oligos (HTO) and antibody-derived tags (ADT): TotalSeqTM-A0301 (BLG-155801), TotalSeqTM-A0303 (BLG-155805), TotalSeqTM-A0304 (BLG-155807), TotalSeq™-A0306 (BLG-155811) TotalSeq™-A0309 (BLG-155817). TotalSeqTM-A0200 CD86 (BLG-105047), TotalSeqTM-A0190 CD274 (BLG-153604).<br>Human monocytes experiments: CD11c (BLG-301623), CD14 (BLG-367115), PD-L1 (BLG-329706), CD86 (BLG-374207), CD1c (BLG-331523), CD209 (BLG-330107), CD11b (BLG-101205), CD141 (BLG-344103).
Validation	All the antibodies are commercial Biolegend antibodies that are in use in many labs. We have tested most of the antibodies using genetic tools. Knocking out the relevant genes and monitoring the protein level by FACS.

Eukaryotic cell lines

Policy information about cell lines and Sex and Gender in Research

Cell line source(s)	We have used B16 cells that originate from ATCC and cells that express OVA protein as indicated in the manuscript
Authentication	The B16 cells without OVA were parchesed from ATCC, we observed and verify the color and morphology of the cells
Mycoplasma contamination	We are testing mycoplasma in all the cell lines in the lab every week. This is critical as we work with innate immune cells. All cell lines are negative to mycoplasma
Commonly misidentified lines (See ICLAC register)	No Commonly misidentified cell lines were used in the study

Animals and other research organisms

Policy information about studies involving animals; ARRIVE guidelines recommended for reporting animal research, and Sex and Gender in Research

Laboratory animals	The following mice strains were purchased from The Jackson Laboratory: C57BL/6-Tg (Tcra Tcrb) 1100Mjb/J (stock# 003831), B6.Cg-Tg (Tcra Tcrb) 425Cbn/J (stock# 004194). Cas9 expressing mice (#026556), BALB/cJ-Cebpb tm1.1Elgaz/J (#032282), C57BL/6J-Tg (Itgax-cre-EGFP) 4097Ach/J (#:007567). Rag-/- mice were obtained from Prof. Yinon Ben Neriah. The C57BL/6 mice (5 weeks) were purchased from Envigo Israel.<br>Mice age were 6-8 weeks and the mouse facility is SPF with restrict 12 hours day/night cycle.
Wild animals	For all the immunological assays we need pure genetic background and used black 6
Reporting on sex	We used females mice in this study.
Field-collected samples	For all the immunological assays we need pure genetic background and used black 6
Ethics oversight	All animal protocols were approved by the joint ethics committee (Institutional Animal Care and Use Committee) of the Hebrew University (Jerusalem, Israel) and Hadassah Medical Center (Jerusalem, Israel). The ethics protocol for mice holding and the experimental procedures are MD-18-15411-5 and MD-19-16071-1.

Note that full information on the approval of the study protocol must also be provided in the manuscript.

ChIP-seq

Data deposition

☐ Confirm that both raw and final processed data have been deposited in a public database such as GEO.

☒ Confirm that you have deposited or provided access to graph files (e.g. BED files) for the called peaks.

Data access links<br>*May remain private before publication.*	Data access revision: https://www.dropbox.com/sh/483b2wmwvdtq3bf/AABz0DfySr-5jWo6LfotsYENa?dl=0
Files in database submission	Cebpb_1.q10_peaks.narrowPeak<br>Cebpb_2.q10_peaks.narrowPeak
Genome browser session<br>(e.g. UCSC)	We are providing all relevant Bigwig files that can be displayed using IGV

Methodology

Replicates	Two technical replicates
Sequencing depth	Cebpb_1 74834876 45219696 18877784 99<br>Input_1 45219696 45219696 16596858 99<br>Cebpb_2 73164692 73164692 15982620 99<br>Input_2 56345352 56345352 20364022 99
Antibodies	Recombinant Anti-CEBP Beta antibody - C-teminal, Abcam, catalog number ab32358, clone name E299, lot number 1000326-2
Peak calling parameters	hisat2 -p 16 -x hisat2_idx/mm10_idx --no-spliced-alignment --very-sensitive -1 Chip-seq_IP_Cebpb_1_S28_R1_001_val_1.fq.gz -2 Chip-seq_IP_Cebpb_1_S28_R2_001_val_2.fq.gz | samtools sort -o Chip-seq_IP_Cebpb_1_S28.bam -<br><br>hisat2 -p 16 -x hisat2_idx/mm10_idx --no-spliced-alignment --very-sensitive -1 Chip-seq_Input_Cebpb_1_S36_R1_001_val_1.fq.gz -2 Chip-seq_Input_Cebpb_1_S36_R2_001_val_2.fq.gz | samtools sort -o Chip-seq_Input_Cebpb_1_S36.bam -<br><br>macs2 callpeak -B -t Chip-seq_IP_Cebpb_2_S31.bam -c Chip-seq_Input_Cebpb_2_S38.bam -f BAM -g mm -n Cebpb_2
Data quality	Paired-end FASTQ files were trimmed to remove adapters and low-quality sequences using trim-galore/0.6.7 and then were aligned to the mm10 reference genome using hisat2/2.2.1 (31375807). picard/2.26.10 MarkDuplicates was used to marke and removed duplicates. Bigwig files were generated using deeptools/3.5.1 (24799436), normalizing by RPKM. Genomic tracks were visualized using The Integrative Genomics Viewer (IGV). Heatmaps were generated using deeptools/3.5.1 and visualized in R using profileplyr/1.14.1.<br>Peaks for each sample were assigned using macs/2.2.7.1 (18798982). A union peak set for all samples was constructed by merging the peaks into a set of high-confidence peaks. ChIPseeker/1.34.1 (25765347) was used for peak annotation. For each comparison, significant peaks were defined as those with a false discovery rate (FDR) lower than 0.05 and fold change threshold > 13 (6094 common peaks between replicates)
Software	trim-galore/0.6.7<br>hisat2/2.2.1<br>macs/2.2.7.1<br>deeptools/3.5.1<br>R4.2.2<br>ChIPseeker/1.34.1<br>HOMER

Flow Cytometry

Plots

Confirm that:

☒ The axis labels state the marker and fluorochrome used (e.g. CD4-FITC).

☐ The axis scales are clearly visible. Include numbers along axes only for bottom left plot of group (a 'group' is an analysis of identical markers).

☒ All plots are contour plots with outliers or pseudocolor plots.

☐ A numerical value for number of cells or percentage (with statistics) is provided.

Methodology

Sample preparation	The cells that were FACS in these study are primary mouse bone marrow cells that were grown in tissue culture plates, and

	then selected or sorted
Instrument	Cells were sorted using BD FACSAria™ III sorter, and analysis was done using cytoflex machine (Beckman Coulter CytoFlex5)
Software	FlowJo software version 10.1r1 was used for FACS analysis.
Cell population abundance	As the experiments are based on invitro culturing, all the cells are relevant.
Gating strategy	We gated on viable cells and then on relevant markers as indicated in the method section.

☒ Tick this box to confirm that a figure exemplifying the gating strategy is provided in the Supplementary Information.
